# Synovial Fluid of Patient With Rheumatoid Arthritis Enhanced Osmotic Sensitivity Through the Cytotoxic Edema Module in Synoviocytes

**DOI:** 10.3389/fcell.2021.700879

**Published:** 2021-08-31

**Authors:** Min Jeong Ji, Hee Jung Ryu, Jeong Hee Hong

**Affiliations:** ^1^Department of Physiology, College of Medicine, Lee Gil Ya Cancer and Diabetes Institute, Gachon University, Incheon, South Korea; ^2^Department of Oral Biology, Yonsei University College of Dentistry, Seoul, South Korea; ^3^Division of Rheumatology, Department of Internal Medicine, Gachon University College of Medicine Gil Medical Center, Incheon, South Korea

**Keywords:** cytotoxic edema, interleukin-6, synovial fluid, NKCC1, aquaporin-1

## Abstract

Rheumatoid arthritis (RA) is an autoimmune disease that causes inflammation of the synovial membrane ultimately leading to permanent damage in the affected joints. For this study, synovial fluids from 16 patients diagnosed with either RA or osteoarthritis (OA) were used to examine volume regulation and cooperative water channels, both of which are involved in the cytotoxic edema identified in RA-fibroblast-like synoviocytes (FLS). The osmolarity and inflammatory cytokine interleukin (IL)-6 of synovial fluids from RA patients were mildly enhanced compared to that from OA patients. RA-FLS demonstrated the enhanced property of regulatory volume increase in response to IL-6 and synovial fluids from RA patients. Although there was no difference in the protein expression of the volume-associated protein sodium–potassium–chloride cotransporter1 (NKCC1), its activity was increased by treatment with IL-6. Membrane localization of NKCC1 was also increased by IL-6 treatment. Additionally, both the protein and membrane expressions of aquaporin-1 were increased in RA-FLS by IL-6 stimulation. The IL-6-mediated enhanced osmotic sensitivity of RA-FLS likely involves NKCC1 and aquaporin-1, which mainly constitute the volume-associated ion transporter and water channel elements. These results suggest that RA-FLS provide enhanced electrolytes and concomitant water movement through NKCC1 and aquaporin-1, thereby inducing cellular swelling ultimately resulting in cytotoxic edema. Attenuation of cytotoxic edema and verification of its related mechanism will provide novel therapeutic approaches to RA treatment within the scope of cytotoxic edema.

## Introduction

The pathology of rheumatoid arthritis (RA) as an autoimmune disease involves joint inflammation, pannus formation, and eventual joint destruction (Nanki et al., 2001). Fibroblast-like synoviocytes (FLS) enter an aggressive state that changes them to have tumor-like characteristics like anchorage-independent growth, resistance to apoptosis, and elevated rates of cellular proliferation in RA due to various proinflammatory mediators (Georganas et al., 2000; Deon et al., 2001). Of those proinflammatory mediators, interleukin (IL)-6 specifically is believed to mediate the pathogenesis of inflammatory arthritis including RA (Kokkonen et al., 2010; Hampel et al., 2013). Several studies suggest that the IL-6 level of synovial fluids revealed differences between the patients with RA and osteoarthritis (OA) (Kokebie et al., 2011; Machado Diaz et al., 2012; Hampel et al., 2013; Elicabe et al., 2017). Although its statistical difference was variable in their studies, the concentration of IL-6 of RA was higher than that of OA. In hypertensive encephalopathy accompanying juvenile RA, it has been reported that enhanced levels of IL-6 are associated with cytotoxic edema (Takano et al., 2002). In addition, IL-6 expression is associated with the development of brain edema in patients with meningiomas (Park et al., 2010).

Change in cellular volume in response to hyper-/hypotonic stimulation is a physiological cellular response. Hypotonic stimulation triggers cell swelling through water influx into the cytosolic area and subsequent elicited regulatory volume decrease (RVD) as a homeostatic reaction. On the contrary, hypertonic stimulation mediates cell shrinkage, which causes regulatory volume increase (RVI) (Huang et al., 2019). This cellular ionic and volume homeostasis is maintained by various ion transporters, of which NKCC1 in particular has been implicated in osmotic cell swelling (Pedersen et al., 2006). NKCC1 is associated with cytotoxic edema in neuronal ischemic stroke and epilepsy (Xu et al., 2017). Although the enhanced IL-6 level in synovial fluids has been suggested and IL-6 is considered to be an important biomarker in RA (Kokebie et al., 2011), the pathogenesis of RA with a focus on cytotoxic edema remains unclear, and the role of NKCC1 and volume homeostasis in FLS remains to be defined.

Synovial fluid is known to contain several immune cells including mast cells, monocytes, synovial lining cells, and inflammatory cytokines, which together represent the degree of inflammation in the affected area (Oliviero et al., 2017). It has been previously shown that synovial fluid is a useful substrate in the diagnosis of joint diseases (Altobelli et al., 2017; Oliviero et al., 2017). In the present study using synovial fluid samples from patients with either RA or OA, we demonstrated IL-6-mediated volume regulation and identified the cooperative water channels involved in cytotoxic edema in RA-FLS. Although RA typically presents with multiple symptoms, attenuation of cytotoxic edema and verification of its related mechanisms will provide new advanced therapeutic approaches for the treatment of RA-related edema.

## Materials and Methods

### Patient Enrollment and Assessment of Clinical Parameters

A total 16 patients (8 patients with RA and 8 patients with knee OA) were enrolled, all of whom had effusion on knee joints. The patients with RA fulfilled the 2010 American College of Rheumatology (ACR)/European League Against Rheumatism (EULAR) classification criteria (Aletaha et al., 2010), and the patients with biological disease-modifying anti-rheumatic drugs (DMARDs) were excluded. The patients with primary knee OA as controls were previously diagnosed based on clinical and radiological examination. In the knee OA group, patients with secondary knee OA due to inflammatory arthritis or metabolic diseases and those with total knee replacements were excluded. All patients underwent medical history and joint examination. In patients with RA, the tender and swollen joint counts were examined, and erythrocyte sedimentation rate (ESR, mm/h) and C-reactive protein (CRP, mg/dl) were measured at enrollment. The status of rheumatoid factor (RF, IU/ml) and anti-citrullinated peptide antibodies (anti-CCP, U/ml) were collected. Disease activities were checked by Disease Activity Score 28-ESR (DAS28-ESR). In patients with knee OA, the severity of knee OA was evaluated by Kellgren–Lawrence (KL) from plain radiographs.

### Synovial Fluid Collection

The synovial fluid (≥10 ml) was obtained by therapeutic arthrocentesis with aseptic procedure. Synovial fluids were aspirated from affected knee joints after acupuncture using a 30-ml syringe with 21-gauge needle. The synovial fluid was then centrifuged at 1,500 rpm for 15 min.

### Reagents

Recombinant human IL-6 (200-06) was purchased from Peprotech (Cranbury, NJ, United States). NKCC1 (ab59791) antibody was purchased from Abcam (Cambridge, United Kingdom). The phosphorylated form of OSR-1 (pOSR-1, Ser325) antibody (07-2273) was purchased from Merck (Darmstadt, Germany). Peroxidase-conjugated β-actin antibody (A3854) was purchased from Sigma-Aldrich (St Louis, MO, United States). OSR-1 (#3729) and IL-6 (#12153) antibodies were purchased from Cell Signaling (Danvers, MA, United States). Aquaporin (AQP)-1 antibody (CSB-PA16085A0Rb) was purchased from CUSABIO (Houston, TX, United States). DAPI fluoromount-G^TM^ mounting solution was purchased from Electron Microscopy Sciences (Electron Microscopy Sciences, Hatfield, PA, United States). 2′-7′-Bis-(carboxyethyl)-5-(and-6)-carboxyfluorescein (BCECF-AM; #0061) was purchased from Teflabs (Austin, TX, United States). Cell volume indicator calcein-AM (C1430) was purchased from Molecular Probes (Eugene, OR, United States). Amiloride (EIPA, Sigma-Aldrich; 1154-25-2) and other chemicals not mentioned here were purchased from Sigma-Aldrich.

### Primary Synoviocytes Culture and HEK293T Cell Culture

Human FLS, isolated from normal synovial tissues (HFLS, 408-05a), RA-FLS (408RA-05a), and HFLS-osteoarthritis (OA) (408OA-05a) cells were purchased from Cell Application, Inc. (San Diego, CA, United States) (Jones et al., 2017; Wang et al., 2017). Cells were maintained in Dulbecco’s modified Eagle’s medium (Invitrogen, Carlsbad, CA, United States; 11995-065) containing 10% fetal bovine serum (FBS, Invitrogen; 16000-044), and 100 U/ml penicillin–streptomycin (Invitrogen; 15140-122) and incubated at 37°C in 5% CO_2_/95% air. When cells reached 80% confluency, the cell culture medium was aspirated, and the cells were washed with Dulbecco’s phosphate-buffered saline (DPBS, Welgene, Daegu, South Korea; LB001-02), followed by treatment with trypsin/ethylene diamine tetraacetic acid (EDTA) for 1 min. Cells were used within five passages for all experiments. Human HEK293T (ATCC^®^, United States; CRL-3216^TM^) cell was maintained by Dulbecco’s modified Eagle’s medium containing 10% fetal bovine serum (FBS) and 100 U/ml penicillin–streptomycin and incubated at 37°C in 5% CO_2_/95% air.

### Enzyme-Linked Immunosorbent Assay for Synovial IL-6 Levels

Synovial fluids were diluted at least 1:1 with the assay diluent of respective ELISA kits, and the level of IL-6 (R&D Systems, Minneapolis, MN, United States; D6050) was measured using the relevant ELISA kits (R&D Systems) according to the manufacturer’s instructions.

### Quantitative RT-PCR

HFLS, OA-FLS, and RA-FLS were seeded in six wells, and IL-6 was treated for 48 h. Total RNA was isolated with RiboEX^TM^ (GeneAll; 301-902). Isopropanol was mixed with isolated RNA on ice for 10 min. All samples were centrifuged at 12,000 *g*, 4°C. Supernatants were removed, and all samples were treated with 70% ethanol. All samples were centrifuged at 7,500 *g* for 10 min at 4°C, and the pellets were air dried. RNA was dissolved in RNase-free water and quantitated using the spectrophotometer ND-1000 (Thermo Fisher Scientific, Waltham, MA, United States). Complementary DNA (cDNA) was amplified with Enzynomics (Daejeon, South Korea) following the manufacturer’s instructions. Quantitative reverse transcription PCR (qRT-PCR) was performed using Power Up^TM^ SYBRTM Green Master Mix (Applied Biosystems^®^, Waltham, MA, United States; A25741), and the cycling protocol was as follows: human GAPDH (forward) GAC AGT CAG CCG CAT CTT CT and (reverse) TGA TGA CCC TTT TGG CTC CC; human NKCC1 (forward) GGA GTG GAA GAC CAC GTG AAA and (reverse) CCC ATA TGT ACA TGG CCA CAG A; human AQP-1 (forward) CAG TGA CCT CAC AGA CCG C and (reverse) TCT ATT TGG GCT TCA TCT CCA CC under UDG activation at 50°C for 2 min, Dual-Lock DNA polymerase at 95°C for 2 min, denaturation at 95°C for 15 s, annealing at 59°C for 15 s, and extension at 72°C for 1 min.

### Osmolarity

Synovial fluids were centrifuged at 1,500 rpm for 15 min to remove cellular debris and liquid phase of supernatant. After heating for 10 min at 37°C, the osmolarity of synovial fluids was measured from OA and RA samples using a Fiske^TM^ 210 Micro-sample Osmometer (Advanced Instruments, Inc., Norwood, MA, United States).

### Analysis of Na^+^–K^+^–2Cl^–^ Co-transporter1 Activity

Na^+^–K^+^–2Cl^–^ co-transporter1 (NKCC1) activity was measured based on the rate of pH_*i*_ decrease induced by intracellular NH_4_^+^ uptake (Evans and Turner, 1997) to determine the electrolyte influx for Na^+^, K^+^, and Cl^–^. To measure transporter activity, FLS cells were attached to a poly-L-lysine-coated coverslip and became adherent during culture using the perfusion-based technique. FLS were attached to coverslips for the measurement of NKCC1 activity. The administration of 20 mM NH_4_Cl in the regular solution (composition is shown in Table 1) induced initial alkalization by NH_3_ diffusion; pH_*i*_ is then decreased due to NH_4_^+^ influx (pulse) as a substitute for K^+^. The pH_*i*_ recovery rate in the second phase following the NH_4_^+^ pulse in the first alkalization phase was defined as the acidification rate (ΔpH_*i*_/s). The slope of acidification rate by pH_*i*_ decrease was considered as the NKCC activity. The emitted fluorescence of the pH indicator BCECF-AM was monitored with a CCD camera (Teledyne Photometrics, Tucson, AZ, United States) attached to an inverted fluorescence microscope (Olympus, Tokyo, Japan) and was analyzed with a MetaFluor system (Molecular Devices, San Jose, CA, United States).

**TABLE 1:** Composition of solution (mM).

**Composition**	**Regular solution**	**Hypertonic-buffered solution**
Sodium chloride (NaCl)	140	140
HEPES	10	10
D-Glucose	10	10
Potassium chloride (KCl)	5	5
Magnesium chloride (MgCl_2_)	1	1
Calcium chloride (CaCl_2_)	1	1
pH	pH 7.4	pH 7.4
Osmolarity	300 mOsm/L	400 mOsm/L

### Measurement of Cellular Volume Changes

The FLS were loaded with the volume indicator calcein-AM (2 μM, Molecular Probes) in the presence of 0.01% pluronic acid (F-127) for 15 min at room temperature. After stabilization of fluorescence, the cells were perfused with HEPES-buffered solution for a minimum of 5 min prior to volume measurement. The calcein-AM was excited at 495 nm, and the emitted fluorescence was measured at 515 nm. The volume of FLS or HEK293T cells was determined using a hypertonic (400 mOsm/L, Table 1) solution (Hwang et al., 2019). Fluorescence images were automatically captured at 1-s intervals using a CCD camera (Q-Imaging, British Columbia, Canada, Retiga 6000) linked to an inverted microscope (Olympus, Tokyo, Japan) and analyzed with the MetaFluor system (Molecular Devices). Each signal was normalized by subtracting the background fluorescence signals to eliminate the raw background signals. RVI was followed by hypertonic stimulation-mediated cell shrinkage. The RVI rate of FLS was determined with slope of RVI increase and compared to that of the non-stimulated control group.

### DNA Transfection Procedure

The human NKCC1 clone (pcDNA3.1) was provided by Dr. Shmuel Muallem at the National Institutes of Health at Bethesda, MD, United States. Plasmid DNAs were incubated in 200 μl of Jet prime buffer (Polyplus, Graffenstaden, France, #B200225) and mixed with 4 μl of the transfection reagent (Polyplus; #21Y0910L1) for 10 min. DNAs were then transferred into HEK293Tcell plates containing culture medium, and all procedures were performed according to the manufacturer’s protocol (Polyplus).

### Confocal Microscopy for Immunostaining

The FLS were cultured on coverslips before being fixed with chilled methanol for 10 min (−20°C). Immunostaining was performed as previously described (Lee et al., 2016) using a 1:100 dilution of NKCC1 and AQP-1 antibodies. Briefly, the bound primary antibodies were detected with fluorescein isothiocyanate (FITC) or Rhodamine (1:100 dilution factor). Coverslips were mounted onto coated glass slides (SuperFrost^*plus*^, Fisher) with DAPI-included fluoromount-G^TM^ (Electron Microscopy Sciences, Hatfield, PA, United States), and images were analyzed using a LSM 700 Zeiss confocal microscope with ZEN software (Fluo-view, Carl Zeiss, Jena, Germany). Images were collected from five separate preparations of FLS, and results were presented as the average from all experiments.

### Western Blotting

The FLS were pre-treated with IL-6 for 48 h. Lysates of FLS were prepared in lysis buffer containing (mM) 20 Tris, 150 NaCl, 2 EDTA, 1% Triton X-100, and a protease inhibitor mixture and were treated as previously described (Lee et al., 2016). Briefly, cellular proteins were denatured in sodium dodecyl sulfate (SDS) sample buffer at 37°C for 30 min. Denatured protein samples (30 μg) were subjected to SDS polyacrylamide gel electrophoresis (SDS-PAGE). Proteins were visualized with NKCC1, OSR-1, pOSR-1, AQP-1, and β-actin antibodies using enhanced luminescence solution (Thermo Fisher Scientific, Waltham, MA, United States) and developed on an X-ray film (Kodak, Tokyo, Japan).

### Statistical Analysis

All experiments were repeated at least three times, and data were expressed as mean ± standard error of the mean (SEM). Our study is based on the fact that IL-6 is one of the main inflammatory cytokines and hypothesize these inflammatory circumstances of RA influence on cellular osmotic modules. To verify this, significant difference of IL-6 between RA and OA was needed. Thus, the sample size was calculated by power 80% and significance 5% (two-sided) on the basis of the article that showed the difference of synovial fluid IL-6 levels between patients with RA and OA (Okamoto et al., 1997). Statistical differences between the mean values from the OA and RA synovial fluid groups and samples were analyzed using Student’s *t*-tests. Statistical significance was defined as p (^∗^*p* < 0.05, ^∗∗^*p* < 0.01, ^∗∗∗^*p* < 0.001).

## Results

### Synovial Fluid IL-6 Level Was Enhanced in RA Patients Compared to OA Patients

**FIGURE 1:**
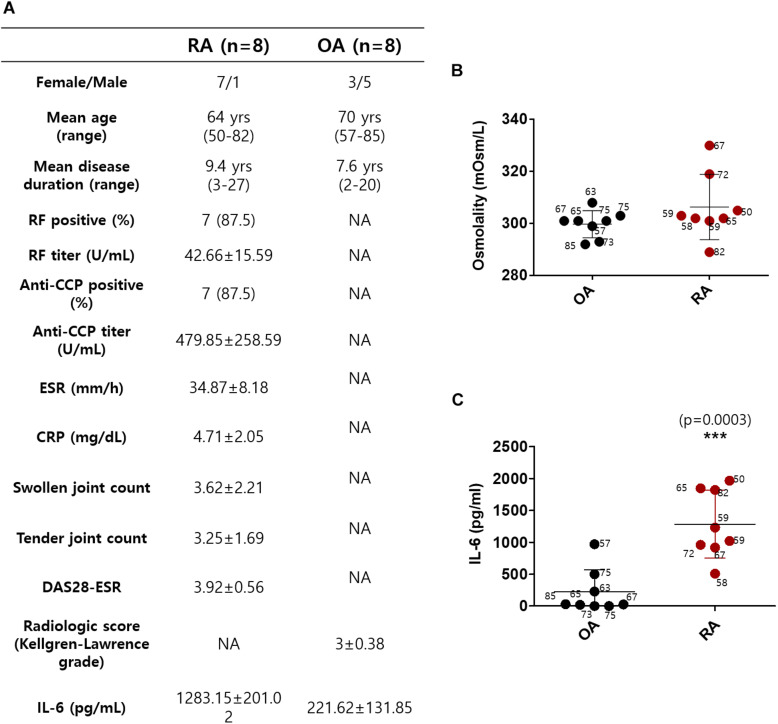
Synovial fluid interleukin (IL)-6 level was enhanced in rheumatoid arthritis (RA) patients compared to osteoarthritis (OA) patients. **(A)** Demographic data of RA patients and OA patients. **(B)** Osmolarity (mOsm/L) of synovial fluids in OA and RA patients. **(C)** Measurement of IL-6 levels in synovial fluids of OA and RA patients using ELISA assay (*n* = 8, ***p < 0.001, *: OA vs RA).

Synovial fluids were obtained from 16 subjects, 8 with RA and 8 with OA (Okamoto et al., 1997). The demographic data of the subjects, including age, gender, and disease duration on the day before surgery, are summarized in Figure 1A. The mean age in the RA group was 64 years; in the OA group, it was 70 years. All of the patients with RA were on the combination therapy with more than one conventional synthetic DMARDs and non-steroidal anti-inflammatory drugs (NSAIDs). The patients with OA were taking NSAIDs or acetaminophen. We determined the osmolarity and the content of IL-6 in synovial fluid to verify the role of IL-6 in cytotoxic edema. The gender of the subjects was not considered. The synovial osmolarity of RA patients was mildly enhanced compared to that of OA patients; however, there was no statistical difference (Figure 1B). The synovial IL-6 levels were enhanced in RA patients compared to OA patients, in agreement with other previous studies (Figure 1C; Kokebie et al., 2011; Machado Diaz et al., 2012; Hampel et al., 2013; Elicabe et al., 2017). There was no statistical difference in represented age of patients between the RA and OA groups (Figures 1B,C).

### IL-6-Stimulated RA-FLS Were Sensitive to Hypertonic Stimulation

**FIGURE 2:**
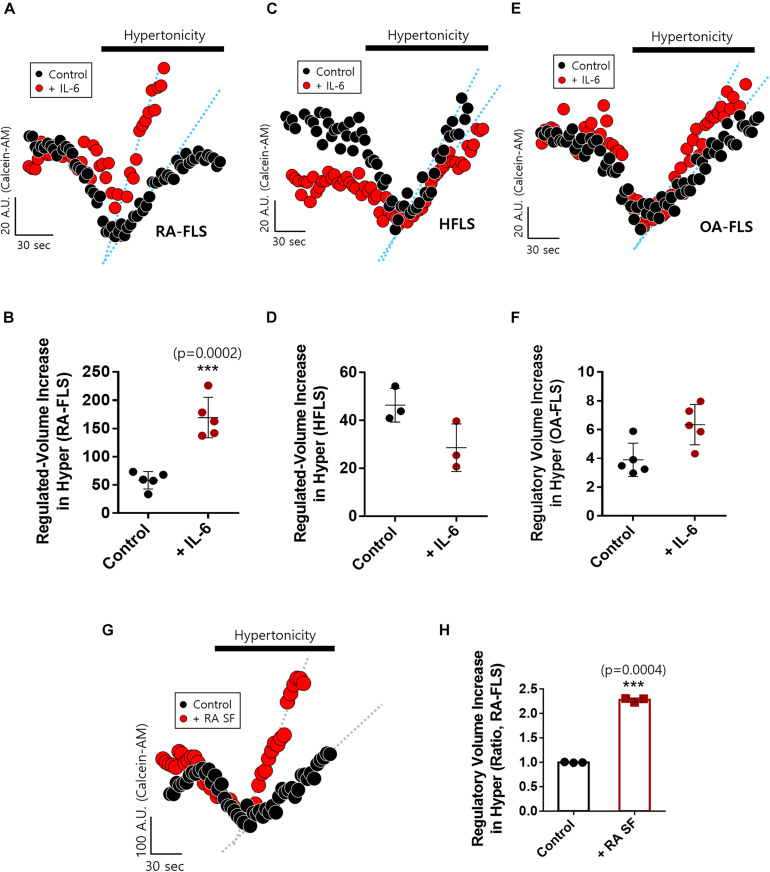
Interleukin (IL)-6-stimulated rheumatoid arthritis fibroblast-like synoviocytes (RA-FLS) were sensitive to hypertonic stimulation. Volume changes in FLS on stimulation with a hypertonic solution (400 mOsm/L) pre-treated with or without 50 ng/ml IL-6 for 48 h in **(A)** RA-FLS, **(C)** human FLS (HFLS), and **(E)** osteoarthritis FLS (OA-FLS). The bars represent the mean ± SEM of volume changes with or without IL-6 treatment in **(B)** RA-FLS, **(D)** HFLS, and **(F)** OA-FLS. [***p < 0.001, *: Control vs + (IL)-6]. **(G)** Volume changes in RA-FLS on stimulation with a hypertonic solution (400 mOsm/L) pre-treated with or without synovial fluid of RA patients for 6 h in a migration assay. **(H)** The bars represent the mean ± SEM of volume changes with or without synovial fluid of RA patients in RA-FLS. Hypertonic solution (hyper) (*n* = 3, ***p < 0.001, *: Control vs + RA SF).

We examined IL-6-mediated osmotic regulation in FLS. The experiment was performed using hyperosmotic stimulation, and the RVI rate was measured. IL-6-stimulated RA-FLS displayed enhanced RVI (Figures 2A,B). RVIs of HFLS and OA-FLS were not statistically different between control and IL-6-stimulated groups (Figures 2C–F). RA synovial fluid contained IL-6 as shown in Figure 1C. Thus, we measured RVI of RA-FLS in the presence of RA synovial fluid. RA synovial fluid-pre-treated RA-FLS enhanced RVI by hypertonic stimulation (Figures 2G,H). These results demonstrate that IL-6-stimulated RA-FLS were more sensitive to hypertonic stimulation than either HFLS or OA-FLS, and RA synovial fluid stimulated enhanced osmotic sensitivity of RA-FLS.

### IL-6 Enhanced NKCC1 Activity in HFLS and RA-FLS

**FIGURE 3:**
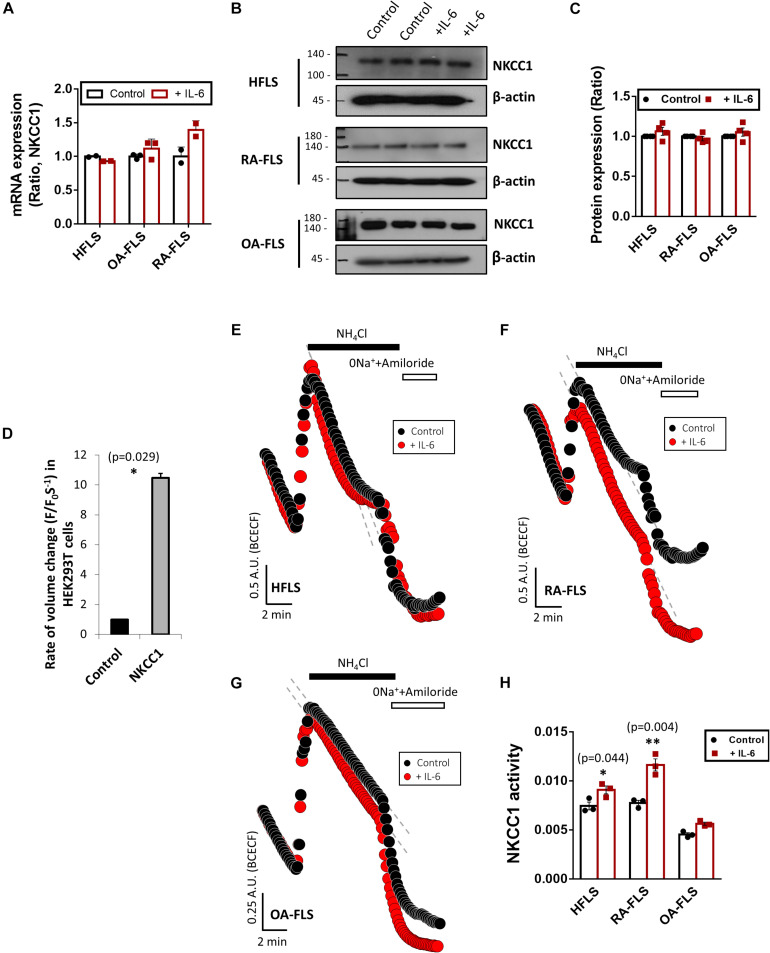
Interleukin (IL)-6 enhanced sodium–potassium–chloride cotransporter 1 (NKCC1) activity in human fibroblast-like synoviocytes (HFLS) and rheumatoid arthritis FLS (RA-FLS). **(A)** mRNA levels and **(B)** protein levels of NKCC1 with pretreatment of 50 ng/ml IL-6 for 48 h in HFLS, OA-FLS, and RA-FLS. β-Actin was used as a loading control. **(C)** Analysis of band intensity of NKCC1 protein levels in HFLS, OA-FLS, and RA-FLS. **(D)** Rate of volume change by hypertonic stimulation in NKCC1-overexpressed HEK293T cells. Results are shown as mean ± SEM (*n* = 3, *p < 0.05, *: Control vs NKCC1). NKCC1 activity due to the 20 mM NH_4_Cl pulse was measured with pretreatment of 50 ng/ml IL-6 in **(E)** HFLS, **(F)** RA-FLS, and **(G)** OA-FLS. **(H)** Analysis of NKCC1 activity. NKCC1 activity of the IL-6-stimulated groups were normalized to control. Results are shown as mean ± SEM [*n* = 3, *p < 0.05, **p < 0.01, *: Control vs + (IL)−6].

RVI caused by osmotic cell swelling requires the involvement of NKCC1 (Hoffmann and Dunham, 1995). Here, we examined the NKCC1 protein expression in IL-6-treated FLS. There were no differences in the messenger RNA (mRNA) and protein expression of NKCC1 of three types of FLS based on IL-6 treatment (Figures 3A–C). To evaluate the role of NKCC1 in volume change, HEK293T cells were made to overexpress NKCC1. Hypertonic stimulation induced a 10-fold higher volume shrinkage in NKCC1-overexpressed HEK293T cells when compared to the wild type of HEK293T cells (control) (Figure 3D). Although the expression level of NKCC1 did not change in both control (HFLS) and RA-FLS based on treatment with IL-6, we determined the NKCC1 activity in the presence of IL-6. NKCC1 activity in HFLS and RA-FLS was enhanced by treatment with IL-6, whereas NKCC1 activity of OA-FLS was mildly enhanced with no statistical difference (Figures 3E–H). NKCC1 activity of RA-FLS was highly responsive to the IL-6 treatment compared with HFLS and OA-FLS.

### IL-6 Stimulated Membranous Expression of NKCC1 and AQP-1

**FIGURE 4:**
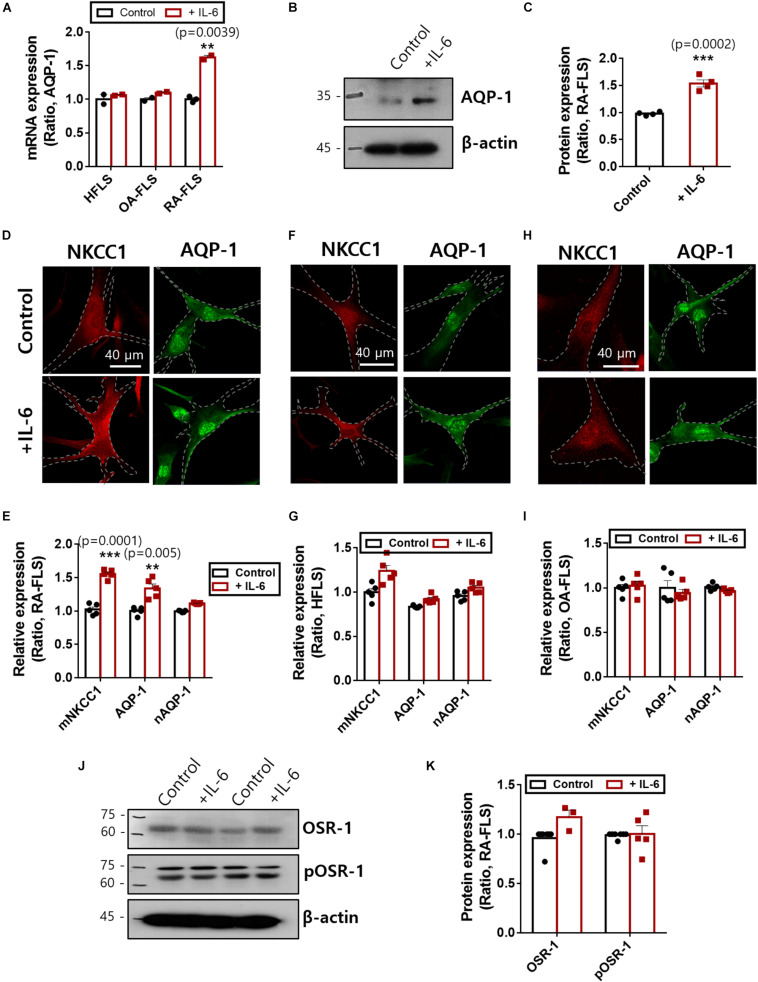
Interleukin (IL)-6 enhanced aquaporin (AQP)-1 expression in rheumatoid arthritis fibroblast-like synoviocytes (RA-FLS). **(A)** mRNA levels [*n* = 3, **p < 0.01, *: Control vs + (IL)−6] and **(B)** protein levels of AQP-1 with pretreatment of 50 ng/ml IL-6 for 48 h in human FLS (HFLS), osteoarthritis FLS (OA-FLS), and RA-FLS. β-Actin was used as a loading control. **(C)** Analysis of band intensity of AQP-1 protein levels in RA-FLS [*n* = 4, ***p < 0.001, *: Control vs + (IL)−6]. Immunolocalization of NKCC1 (red) and AQP-1 (green) with treatment of 50 ng/ml IL-6 in **(D)** RA-FLS, **(F)** HFLS, and **(H)** OA-FLS. Analysis of membranous intensity of sodium–potassium–chloride cotransporter1 (NKCC1) and AQP-1 and nuclear staining of AQP-1 in **(E)** RA-FLS [*n* = 5, **p < 0.01 and ***p < 0.001, *: Control vs + (IL)−6], **(G)** HFLS, and **(I)** OA-FLS. **(J)** Protein levels of OSR-1 and pOSR-1 with treatment of 50 ng/ml IL-6 for 48 h of RA-FLS. β-Actin was used as a loading control. **(K)** Analysis of band intensity of OSR-1 and pOSR-1 in RA-FLS.

NKCC1 recruits water channels to maintain volume homeostasis. Among those water channels, AQP-1 was expressed in FLS and revealed enhanced expression in RA (Trujillo et al., 2004; Mu et al., 2020). We confirmed that treatment with IL-6 enhanced mRNA and protein expression of AQP-1 in RA-FLS (Figures 4A–C). Additionally, NKCC1 and AQP-1 were stained with and without IL-6 treatment. We found recruitment of NKCC1 on the plasma membrane in IL-6-treated RA-FLS (Figure 4D). We measured the region of interest of the membrane and cytosolic area. Nuclear immunoreactivity of AQP-1 was also observed. Membranous expression of NKCC1 and AQP-1 was enhanced in RA-FLS (Figures 4D,E), whereas those of HFLS and OA-FLS had no difference by IL-6 stimulation (Figures 4F–I and Supplementary Material). No changes in nuclear staining of AQP-1 were observed in the three types of FLS (Figures 4E,G,I). Cellular volume and osmotic regulation were mediated by the involvement of OSR-1 (Sengupta et al., 2013). Enhanced hypertonic sensitivity and osmotic regulation by IL-6 may induce the phosphorylation of OSR-1. Thus, we determined the protein expression of OSR-1 in the presence of IL-6. The phosphorylated and total forms of OSR-1 showed no change with IL-6 treatment after 48 h (Figures 4J,K). These results demonstrate that enhanced NKCC1 and AQP-1 expression was a complementary process working against osmotic changes caused by IL-6-stimulated ion flux; these mechanisms operated without changing the expression of the osmolarity-sensitive protein OSR-1.

### RA Synovial Fluid Enhanced NKCC1 Activity, and IL-6 Antibody Blocked the Effect of RA Synovial Fluid in RA-FLS

**FIGURE 5:**
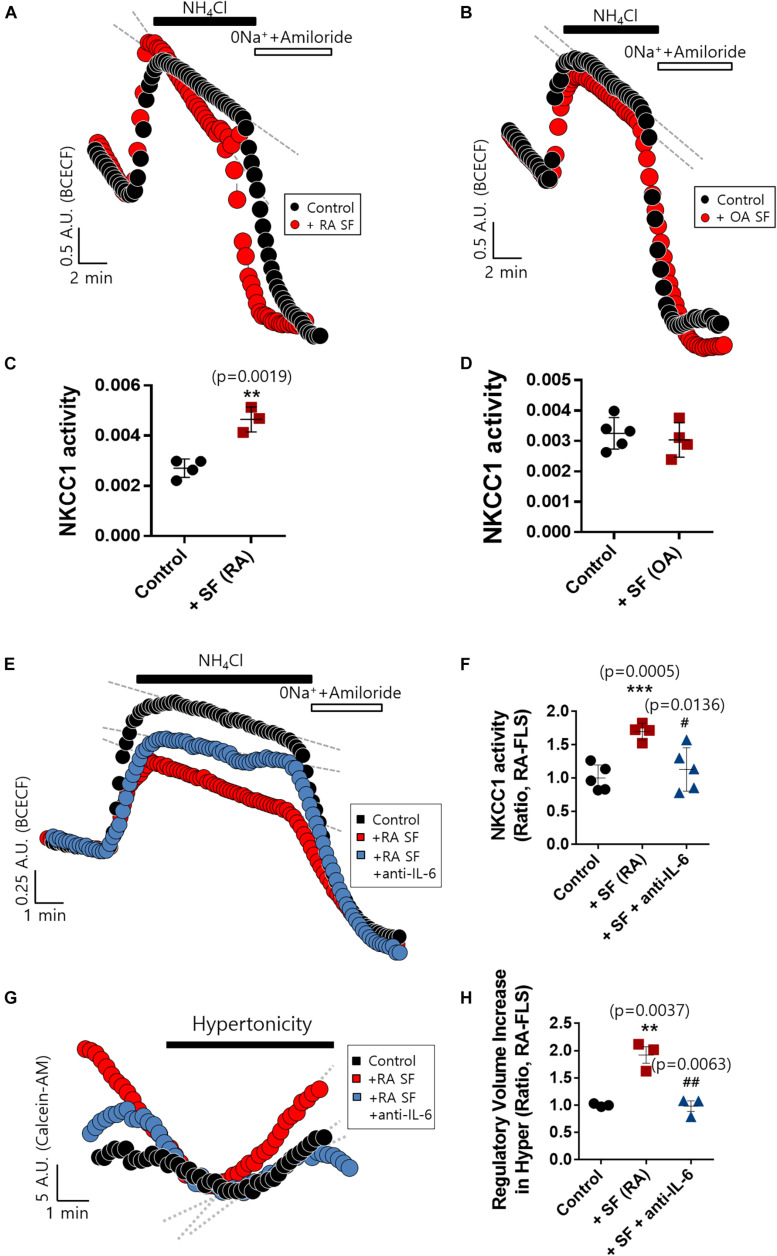
Rheumatoid arthritis (RA) synovial fluid enhanced sodium–potassium–chloride cotransporter1 (NKCC1) activity and interleukin (IL)-6 antibody blocked the effect of RA synovial fluid in RA fibroblast-like synoviocytes (RA-FLS). NKCC1 activity was measured using the membrane of a Boyden Transwell chamber with pretreatment with **(A)** RA and **(B)** osteoarthritis (OA) synovial fluid for 6 h in RA-FLS. The bars represent the mean ± SEM of NKCC1 activity with or without **(C)** RA [*n* = 4, **p < 0.01, *: Control vs + SF (RA)] and **(D)** OA synovial fluid in RA-FLS. **(E)** NKCC1 activity by the stimulation of RA synovial fluid with or without 5 μg/ml anti-IL-6 antibody for 6 h in RA-FLS on the membrane of Boyden Transwell chamber. **(F)** The bars represent the mean ± SEM of NKCC1 activity [*n* = 5, ***p < 0.001, and ^#^*p* < 0.05, *: Control vs + SF (RA), #: + SF (RA) vs + SF + anti-(IL)−6]. **(G)** Volume changes in RA-FLS on stimulation with a hypertonic solution (400 mOsm/L). RA synovial fluid was pre-treated with 5 μg/ml anti-IL-6 antibody for 1 h and then stimulated to RA-FLS on the membrane of Boyden transwell chamber for 6 h. **(H)** The bars represent the mean ± SEM of volume changes. Hypertonic solution (hyper) [*n* = 3, **p < 0.01 and ^##^*p* < 0.01, *: Control vs + SF (RA), #: + SF (RA) vs + SF + anti-(IL)−6].

The concentration of IL-6 in RA synovial fluid was 6-fold higher than that of OA synovial fluid as shown in Figure 1C. We determined whether the synovial fluid of RA patients directly enhanced NKCC1 activity. Direct stimulation with synovial fluid on FLS was technically limited due to its viscosity and turbidity. Thus, RA-FLS were stimulated with synovial fluid in the bottom chamber of the Boyden Transwell system to prevent these obstacles to imaging NKCC1 activity. Three synovial fluids of RA and OA were randomly selected, respectively. Stimulation with RA synovial fluid enhanced NKCC1 activity in RA-FLS (Figures 5A,C), whereas OA synovial fluid revealed no differences between the control and stimulated group (Figures 5B,D). In addition, we hypothesized that RA synovial fluids, including IL-6, activated FLS. To remove the effect of IL-6 in RA synovial fluid, IL-6 antibody was pre-treated in RA synovial fluid. Enhanced NKCC activity and RVI by RA synovial fluid were diminished by the pretreatment of IL-6 antibody in RA synovial fluid (Figures 5E–H). These results confirmed that RA synovial fluid enhanced NKCC1 activity, and the treatment of IL-6 antibody blocked the effects of RA synovial fluid on the modulation of NKCC activity and RVI in RA-FLS.

## Discussion

This study addressed for the first time whether RA synovial fluids or IL-6 would enhance the protein expression and activity of NKCC1 and complementary AQP-1 in RA-FLS. Osmotic changes in synovial fluids, such as the mildly hypertonic condition caused by RA, revealed more sensitive volume changes in RA-FLS than in OA-FLS or HFLS. Our study provided the pathophysiological role of NKCC1 and AQP-1 as inflammation-linked joint edema modules in the pathogenesis of RA patients.

Joint swelling is considered to be one of the 2010 ACR–EULAR classification criteria for RA diagnosis (Aletaha et al., 2010). We hypothesized that the osmotic change of synovium is associated with molecular edema module, such as NKCC1 or OSR-1 in FLS. RVI caused by hyperosmotic stimulation is mediated by ion influx with accompanying water and phosphorylation of NKCC1 and activation of OSR-1 (Alessi et al., 2014). Our results suggest that RA-FLS possessed high potential of RVI through the convergent regulation of NKCC1 and AQP-1 in response to proinflammatory cytokines within synovial fluid, including IL-6. It has been suggested that levels of IL-6 are associated with cytotoxic edema (Takano et al., 2002).

**FIGURE 6:**
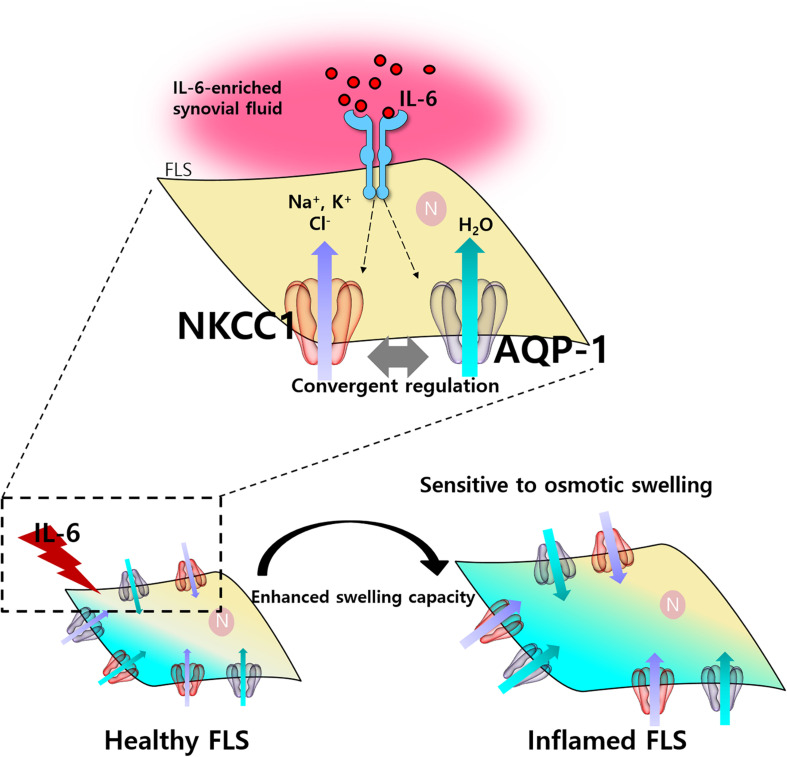
Schematic illustration of osmotic change by interleukin (IL)-6 through sodium–potassium–chloride cotransporter1 (NKCC1) and aquaporin (AQP)-1 in fibroblast-like synoviocytes (FLS). IL-6-induced activation of NKCC1 and AQP-1 in FLS. Activation of NKCC1 and AQP-1 by IL-6/IL-6-enriched synovial fluid resulted in the influx of solute and water causing the sensitivity to hyperosmotic stimulation.

RA synovial fluids were observed to have a relatively high cytokine level compared to OA samples in Figure 1A. Many reports used RA/OA comparison based on current knowledge as shown in Plebanczyk et al. (2019). For comparison between healthy control and RA patient, it is difficult to obtain synovial fluid from healthy persons because it cannot be aspirated sufficiently for our experiment (at least more than 10 ml). Related to the joints from healthy persons who have surgery due to trauma, they actually did not mean complete healthy tissues because they have complex healing process including inflammation, although enough synovial fluid could be collected. Several inflammatory cytokines including IL-6 have been found in synovial fluids from RA patients. The profile of several inflammatory cytokines, such as IL-1, IL-6, IL-8, IL-18, and IL-29 in synovial fluids was associated with RA (Kaneko et al., 2000; Shao et al., 2009; Kokebie et al., 2011; Wang et al., 2012). The role of IL-6 on RA was clearly shown in IL-6-mediated osmotic regulation and enhanced NKCC activity in RA-FLS and concentration of IL-6 in RA synovial fluids. The administration of IL-6 antibody on RA synovial fluids also revealed the reduced osmotic responsiveness and NKCC1 activity in RA-FLS as shown in Figures 5E–H. However, therapeutic approach for the blockade of IL-6 or IL-6 receptor should require careful consideration. The IL-6 receptor blocker, tocilizumab, has enhanced serum IL-6 level (Nishimoto et al., 2008). Tocilizumab-associated enhanced IL-6 level may enhance cytotoxic edema module as addressed by our results. Thus, Figure 6 represents a schematic illustration of this osmotic regulation mechanism in RA-FLS.

Additionally, we explored the mechanisms of osmotic regulation in inflammatory mediator-induced cytotoxic edema, which also occurred in the joint area. Generally, edema is classified in the cerebral region and divided as vasogenic, cytotoxic, or interstitial edema (Bhardwaj, 2007). We hypothesized, as mentioned in the beginning of this paragraph, that FLS could also be elements in cytotoxic edema just as neurons and endothelial cells are in the cerebral regions. In this study, we proposed the concept of synovial ischemia to represent the swelling of FLS caused by the influx of electrolytes and water similar to that seen in cerebral ischemia (Dijkstra et al., 2016). The current study reveals that AQP-1 is clearly implicated in the development of joint edema caused by FLS swelling in the inflammatory synovium, similar to AQP-4 mediated astrocyte swelling in brain edema (Rao et al., 2011). In addition, ischemic heart injury is mediated by the involvement of NKCC1 (Ramasamy et al., 2001). IL-6 activates NKCC1 activity in cardiac tissue (Ji et al., 2020) and induces NKCC1 phosphorylation and Cl^–^ accumulation in sensory neurons (Pieraut et al., 2011). The enrollment of NKCC1 is also associated with cellular volume change, membrane permeability, and subsequent change in water content in the brain (Gong et al., 2021). Additionally, the multiple roles of IL-6 spread throughout both inflammatory and metabolic processes, making contributions to both of these vital pathways (Jones and Jenkins, 2018). The signaling axis of IL-6 is considered the central process of inflammation and autoimmunity in various tissues including pulmonary, cardiac, and joints (Hirano et al., 1987; Tanaka et al., 2014; Ji et al., 2020; Wang and He, 2020; Gong et al., 2021). IL-6 induces enhanced production of vascular endothelial growth factor, resulting in enhanced angiogenesis and vascular permeability (Nakahara et al., 2003) and induced inflammatory signal of immune cells including T helper 17 (Th17) cells (Swaak et al., 1988; Bettelli et al., 2006; Yue et al., 2010). Th17 activation and arthritis activity, such as joint swelling of CIA mice were inhibited by the treatment of anti-IL-6R antibody (Fujimoto et al., 2008). In addition, joint inflammation was suppressed in IL-6-deficient mice (Nowell et al., 2003). In this study, our experimental evidence directly provide that the enhanced IL-6 level of RA patients compared to that of OA can be a switch to turn on the intracellular IL-6 signaling, such as enhanced cytotoxic edema modules NKCC1 and AQP-1 in RA. Although there are various degrees, these results implicate the pathophysiological role of inflamed synovial fluids in the pathogenesis of RA.

We expected the enhanced expression of NKCC1 in the presence of IL-6 or IL-6-enriched synovial fluid; however, the mRNA and protein expression was not changed. Membrane recruitment of transporters and channels was functionally working without increased net protein expression. In addition, RVI or RVD occurs in response to osmotic changes (O’Brien et al., 1993). We hypothesized that if osmolarity-sensitive proteins, such as NKCC1 are recruited to the plasma membrane, our experimental stimulation of hyperosmolar solution mediated cellular volume change. Our experiment was performed in a relatively high osmolarity of 400 mOsm/L. We suggest that these data did not mean that synovial fluids were hyperosmolar. The RVI tool was used in the current study to verify the volume regulation of osmotic-responsive protein, such as NKCC1. Our results suggest that enhanced membrane recruitment of NKCC1 and combined water channels provide favorable circumstances for the homeostatic response to hyperosmotic stimulation in patients with inflamed synovial fluid. We propose that blocking the osmotic sensitivity of FLS may represent a new therapeutic strategy not only for cytotoxic edema in RA but also for other osmotic dysfunctions including brain and cardiac edemas.

## Data Availability Statement

The raw data supporting the conclusions of this article will be made available by the authors, without undue reservation.

## Ethics Statement

This study was conducted in accordance with the Declaration of Helsinki and was approved by the Institutional Review Board of Gil Medical Center (GAIRB-2019-018). Written informed consent was obtained from each individual.

## Author Contributions

JH and MJ contributed to the conception and design, acquisition, or analysis and interpretation of data. MJ and HR made the article drafts, acquired the data, and performed critical revisions for the important intellectual content. HR collected the synovial fluid from the human subjects. JH drew all schematics. JH and HR contributed to the final approval of the version to be published and agreed to be held accountable for all aspects of the work, ensuring that questions related to the accuracy or integrity of any part of the work are appropriately investigated and resolved. All authors contributed to the article and approved the submitted version.

## Conflict of Interest

The authors declare that the research was conducted in the absence of any commercial or financial relationships that could be construed as a potential conflict of interest.

## Publisher’s Note

All claims expressed in this article are solely those of the authors and do not necessarily represent those of their affiliated organizations, or those of the publisher, the editors and the reviewers. Any product that may be evaluated in this article, or claim that may be made by its manufacturer, is not guaranteed or endorsed by the publisher.
