# Supplementary material for: Synovial Fluid of Patient With Rheumatoid Arthritis Enhanced Osmotic Sensitivity Through the Cytotoxic Edema Module in Synoviocytes
Source: Front Cell Dev Biol. 2021 Aug 31;9:700879. doi: 10.3389/fcell.2021.700879 (PMC8438158; doi:10.3389/fcell.2021.700879)

Figure 3. Ji et al.

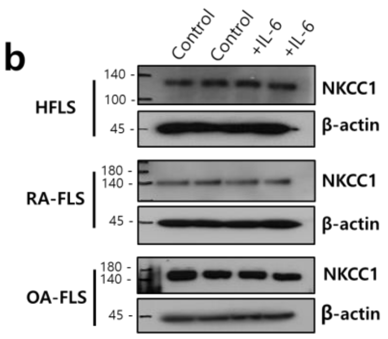

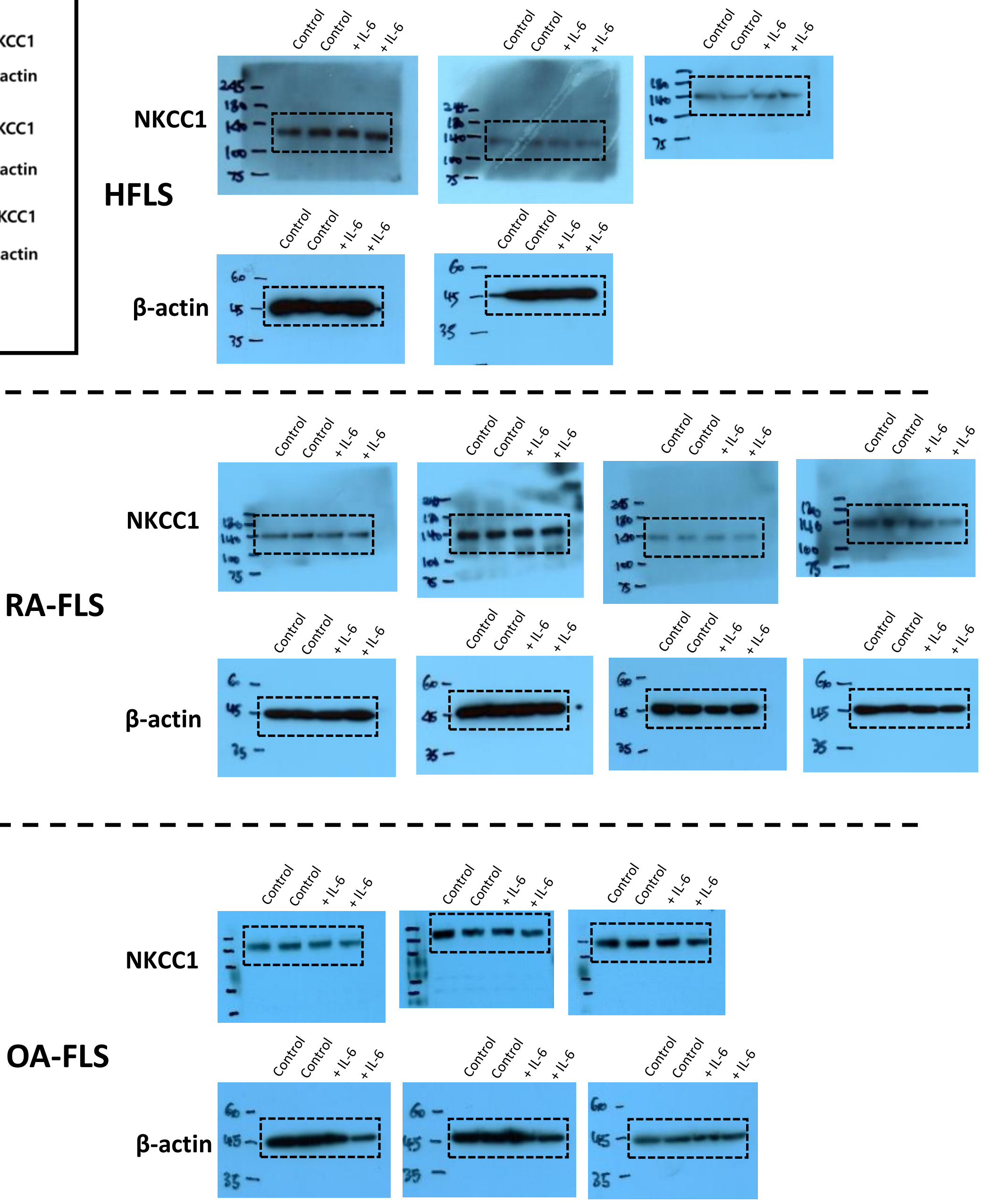

Figure 4. Ji et al.

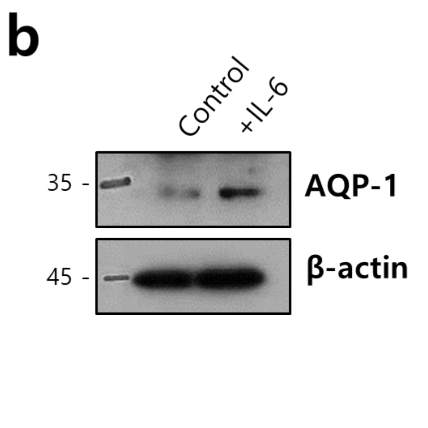

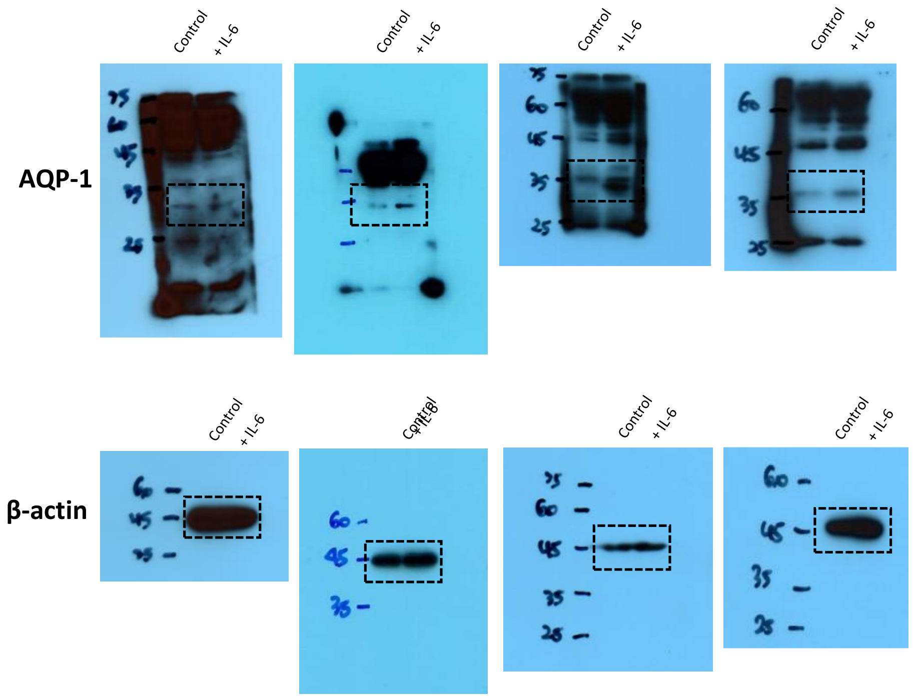

Figure 4. Ji et al.

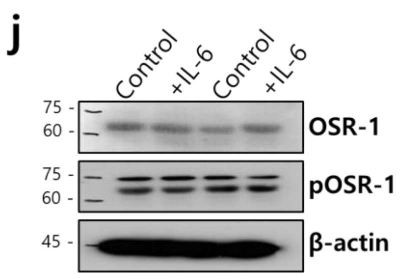

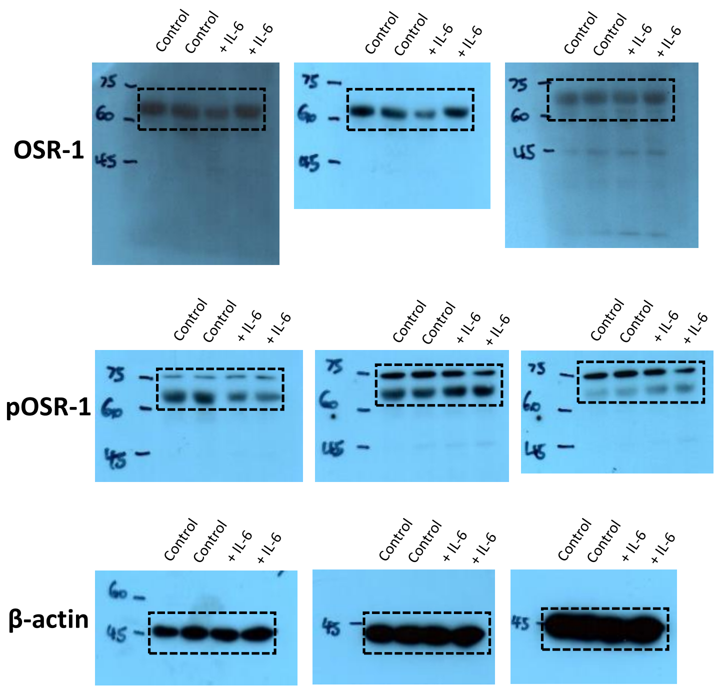

Figure 4. Ji et al.

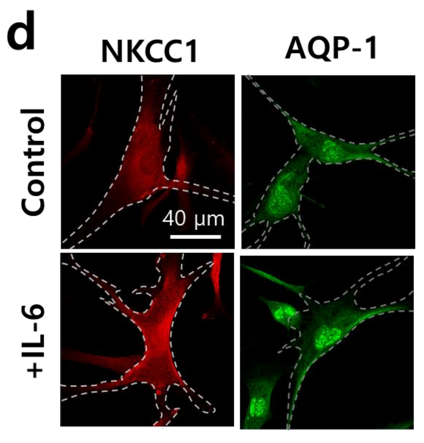

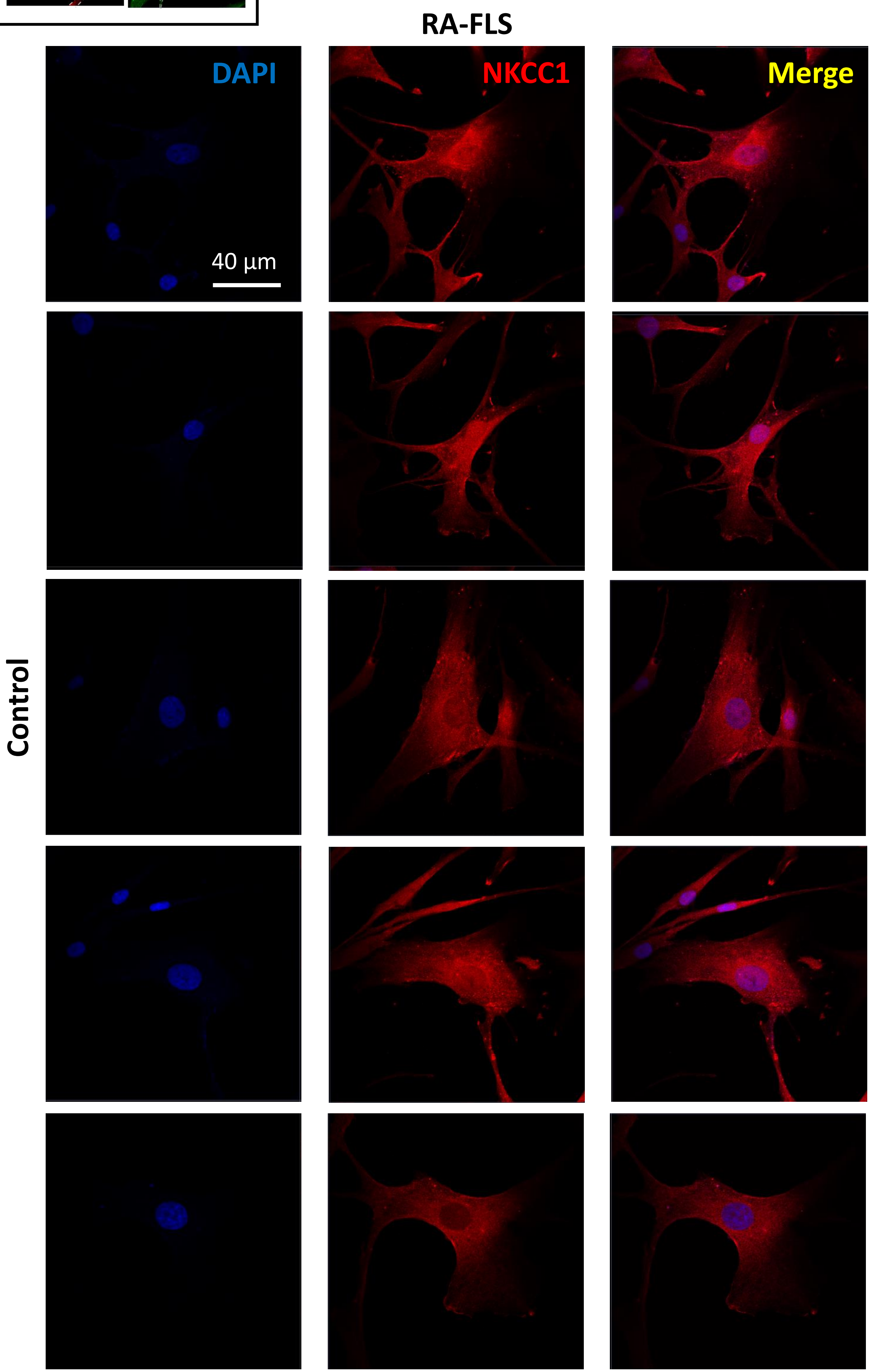

Figure 4. Ji et al.

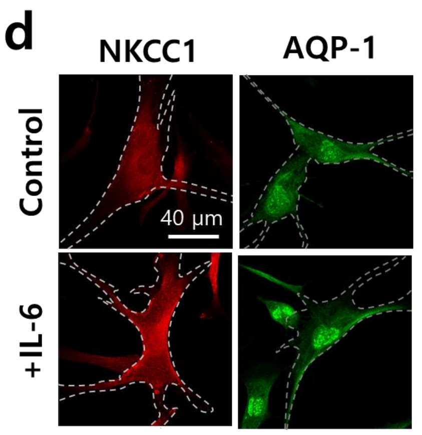

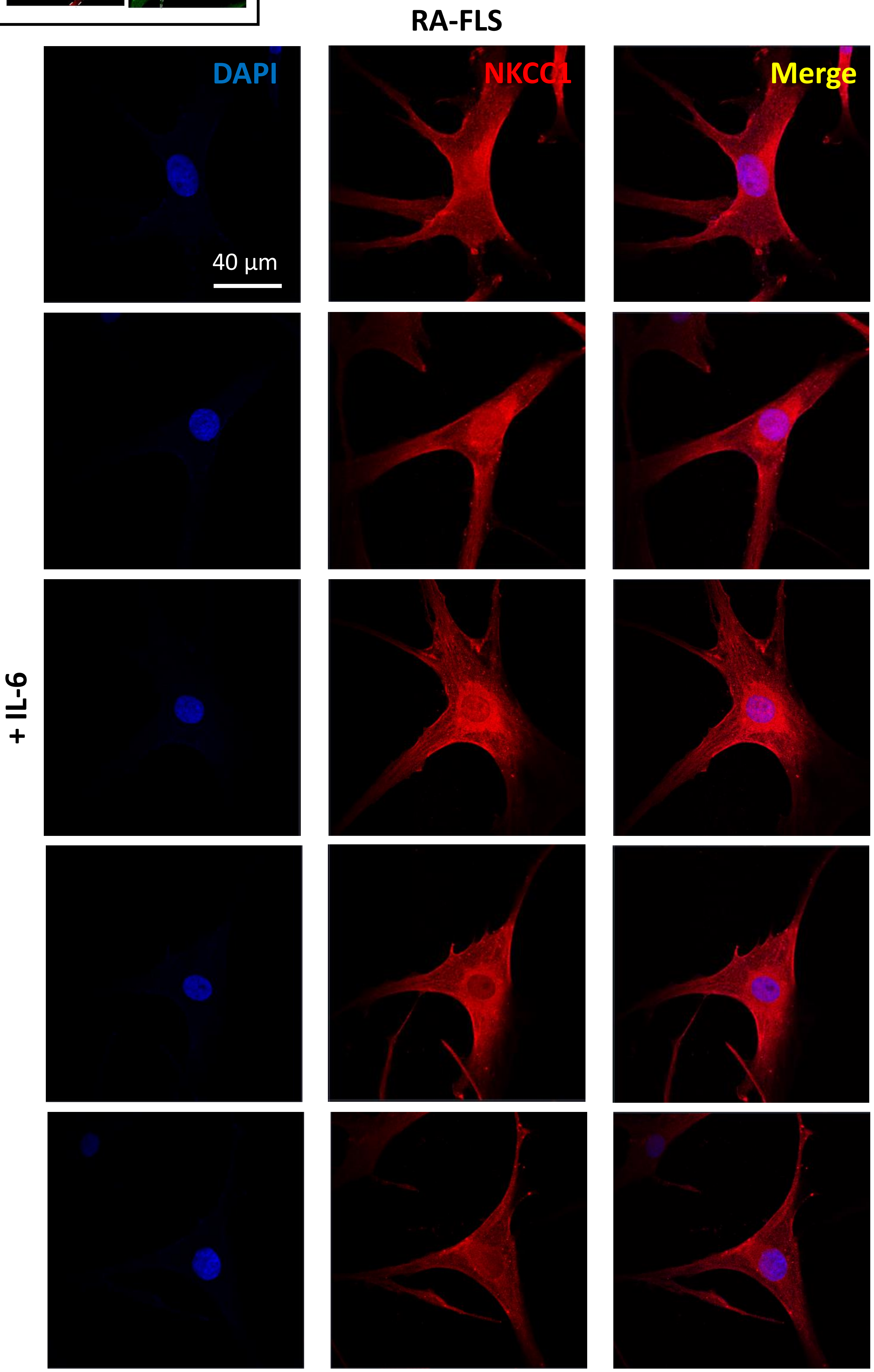

Figure 4. Ji et al.

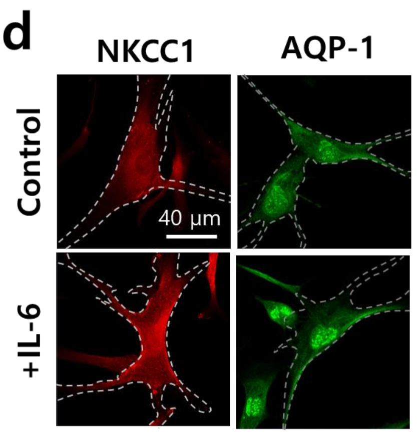

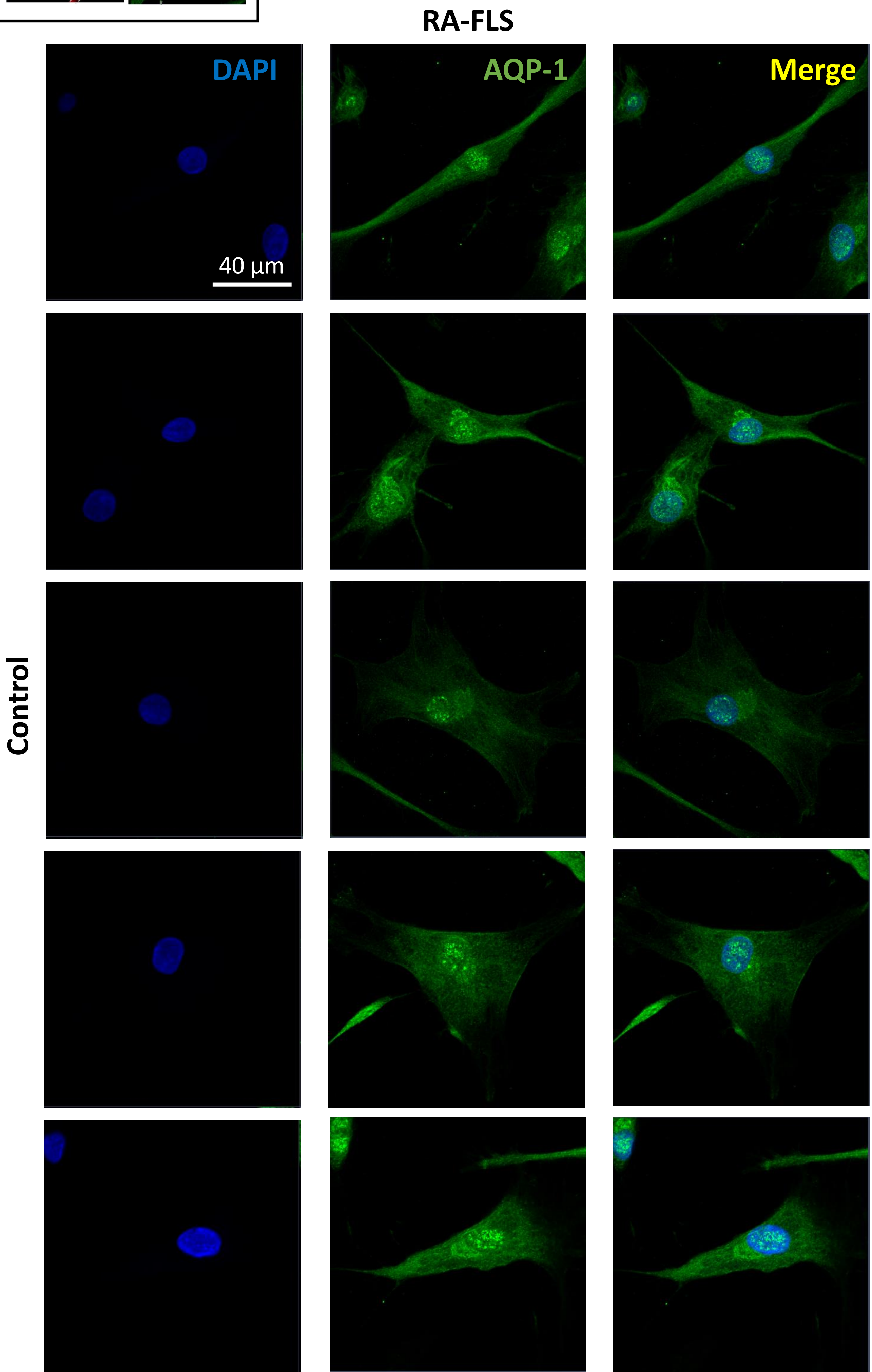

Figure 4. Ji et al.

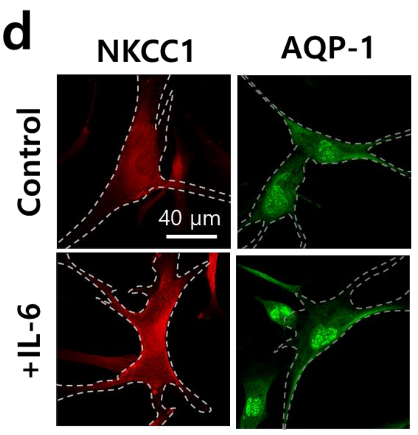

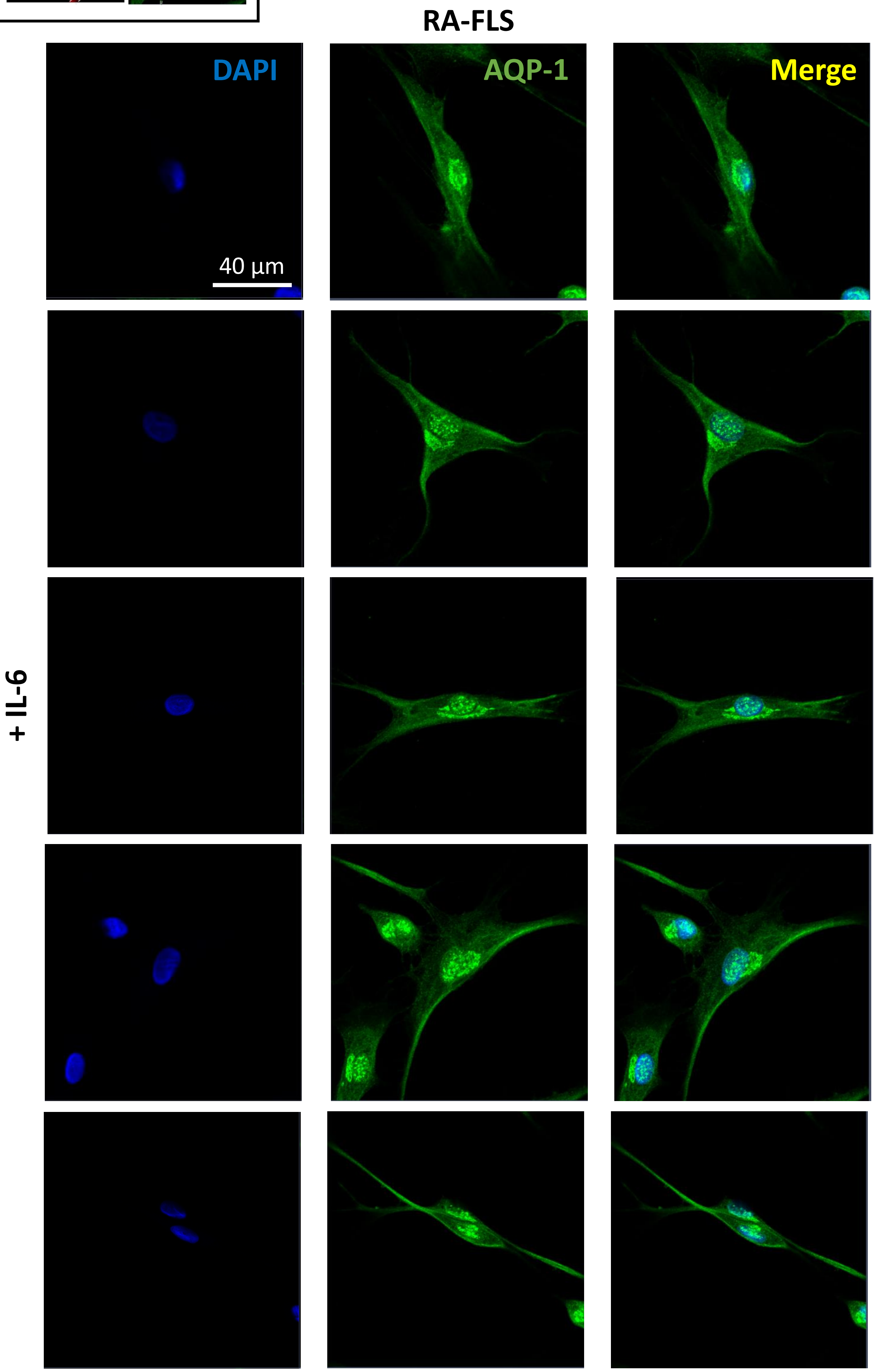

Figure 4. Ji et al.

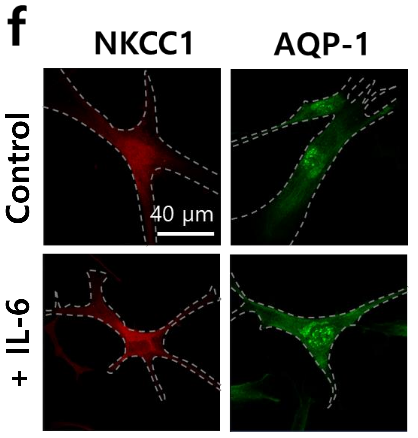

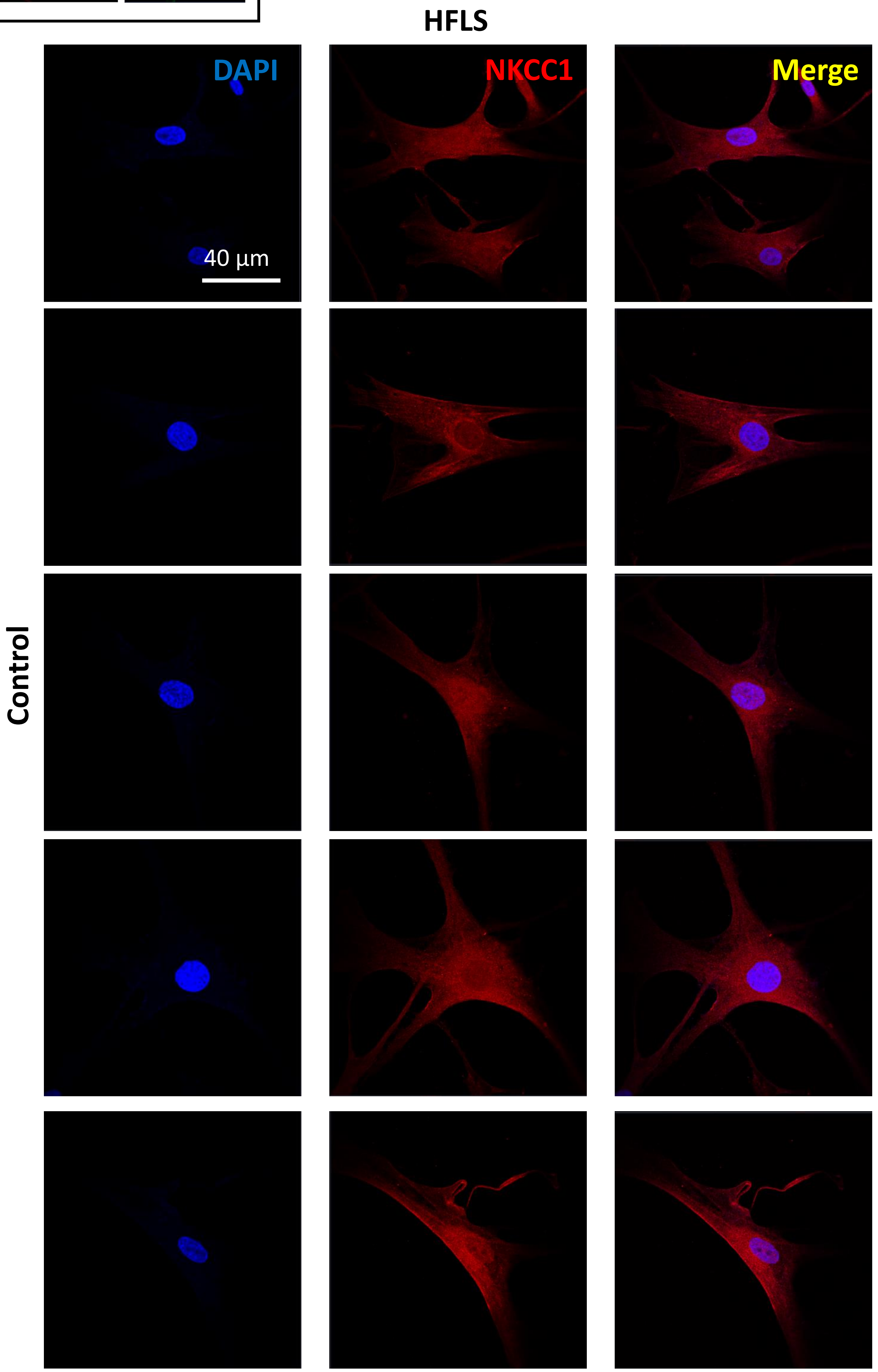

Figure 4. Ji et al.

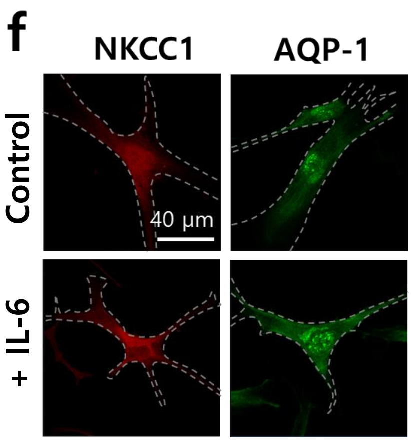

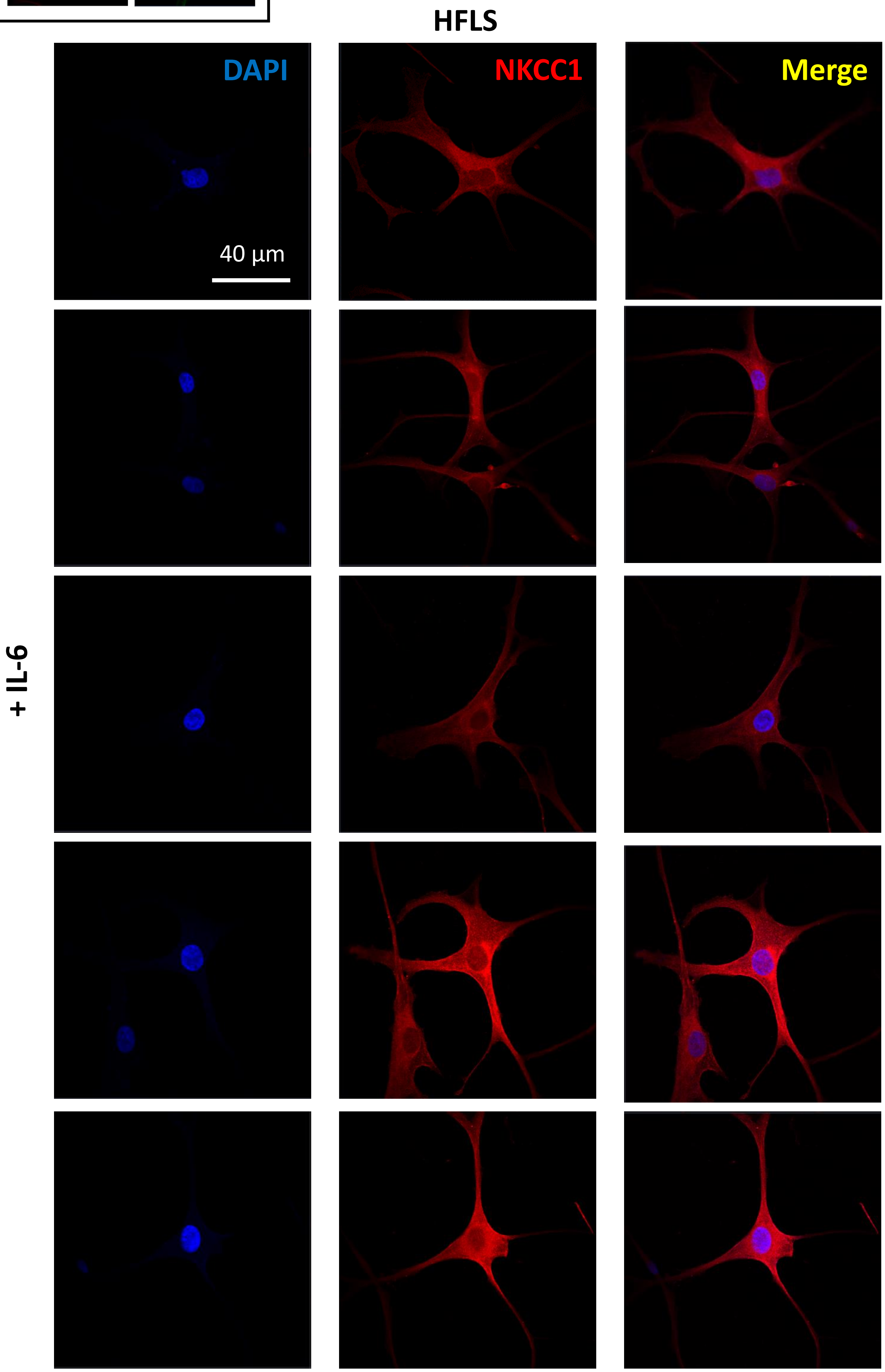

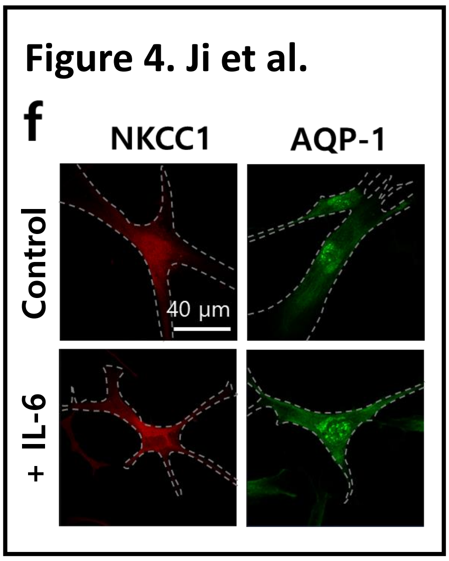
Figure 4. Ji et al.
f
NKCC1
AQP-1
Control
+ IL-6
40 µm

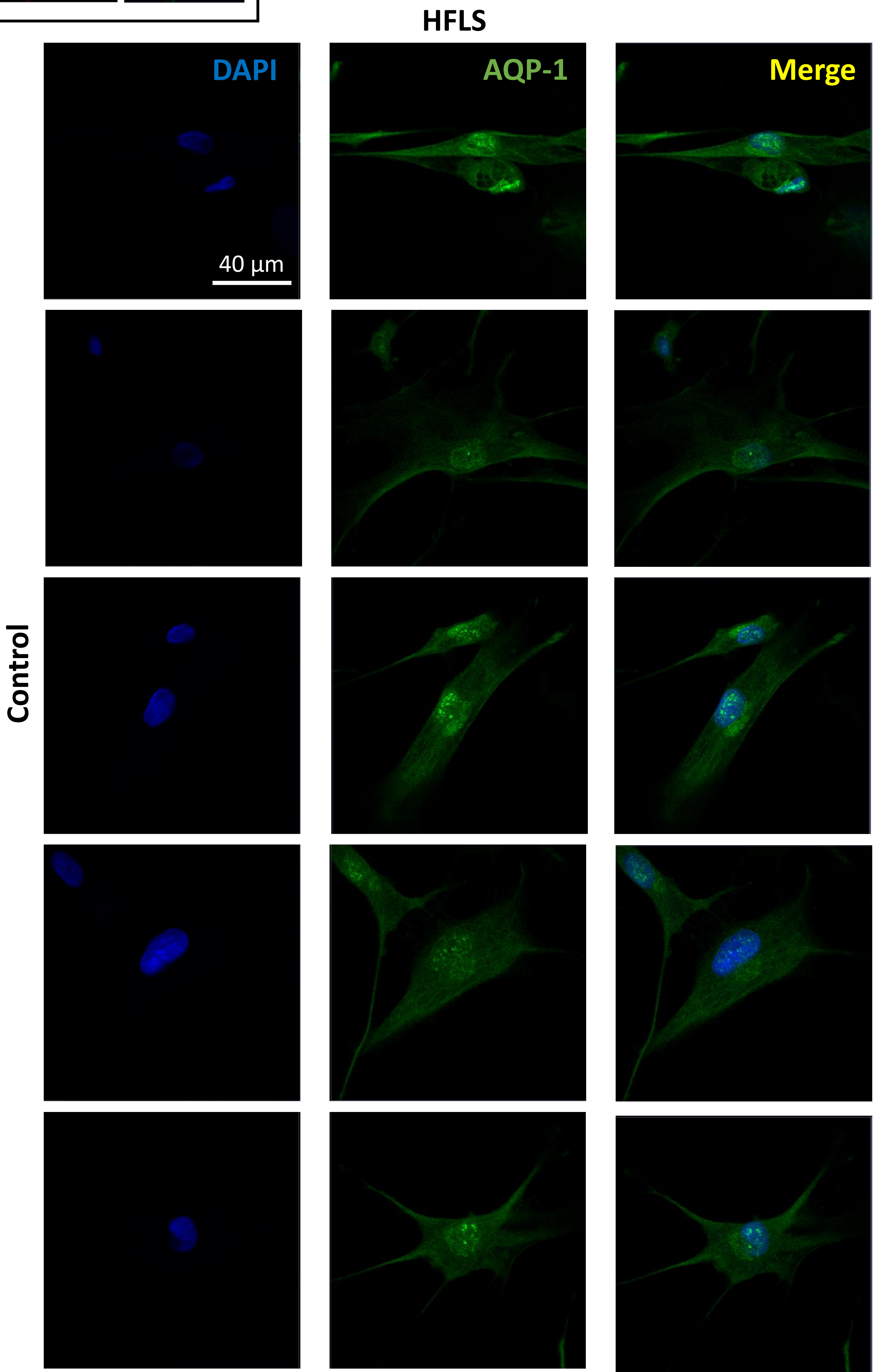
HFLS
DAPI
AQP-1
Merge
Control
40 µm

Figure 4. Ji et al.

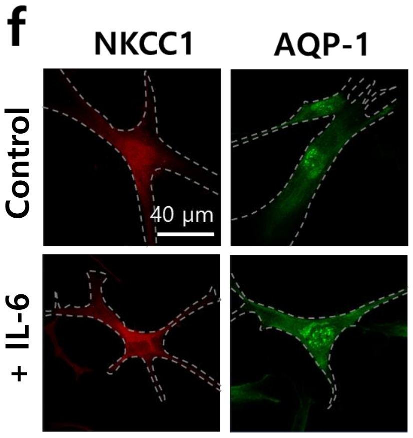

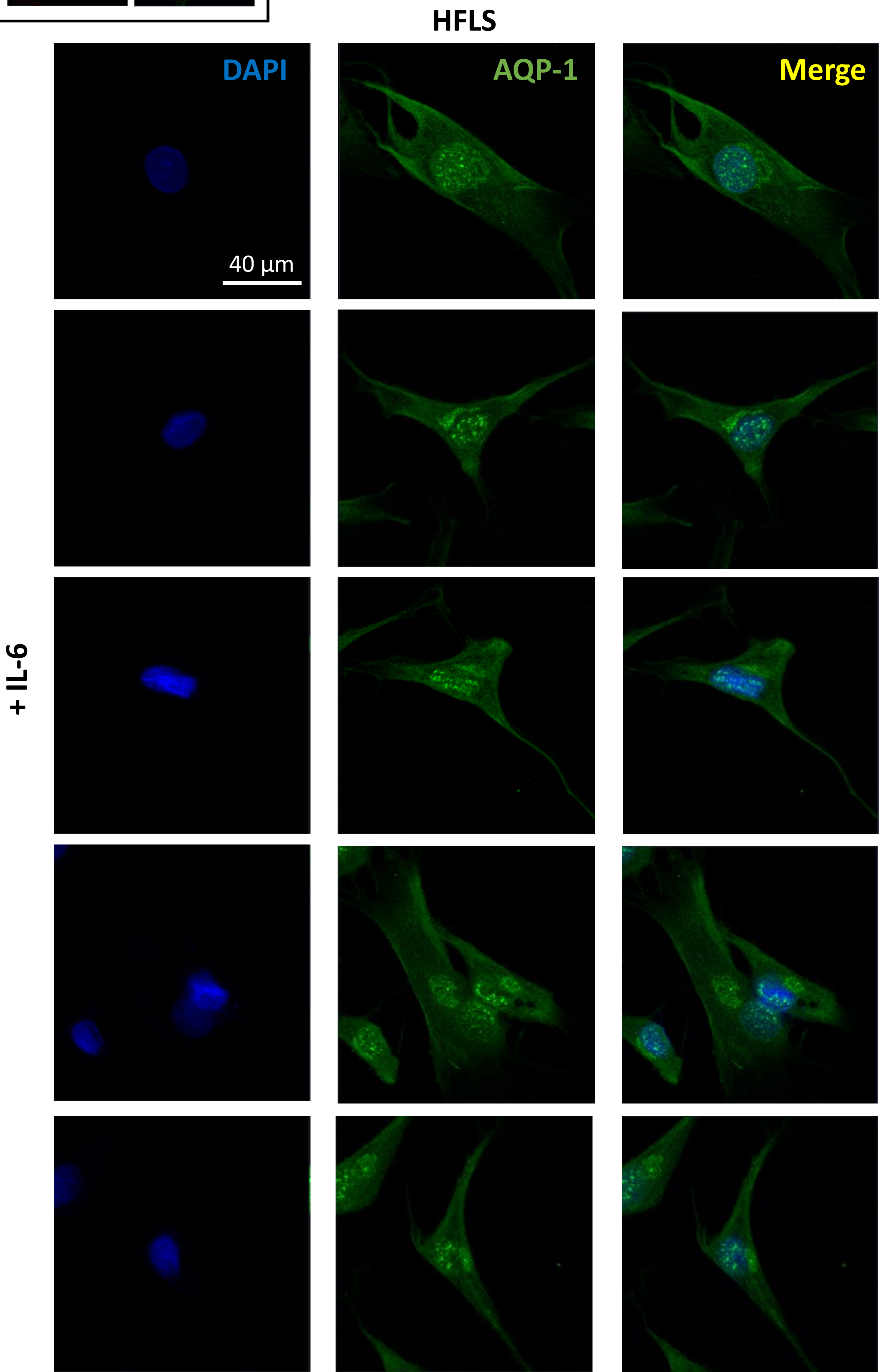

Figure 4. Ji et al.

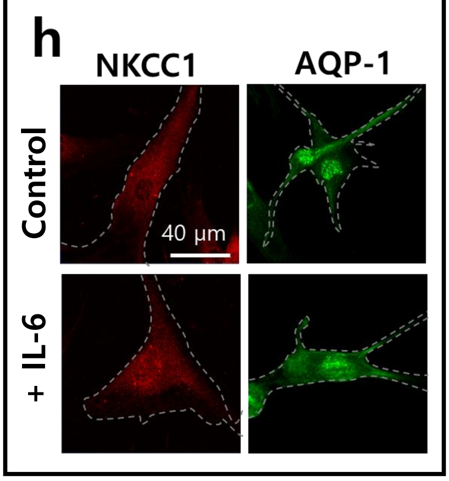

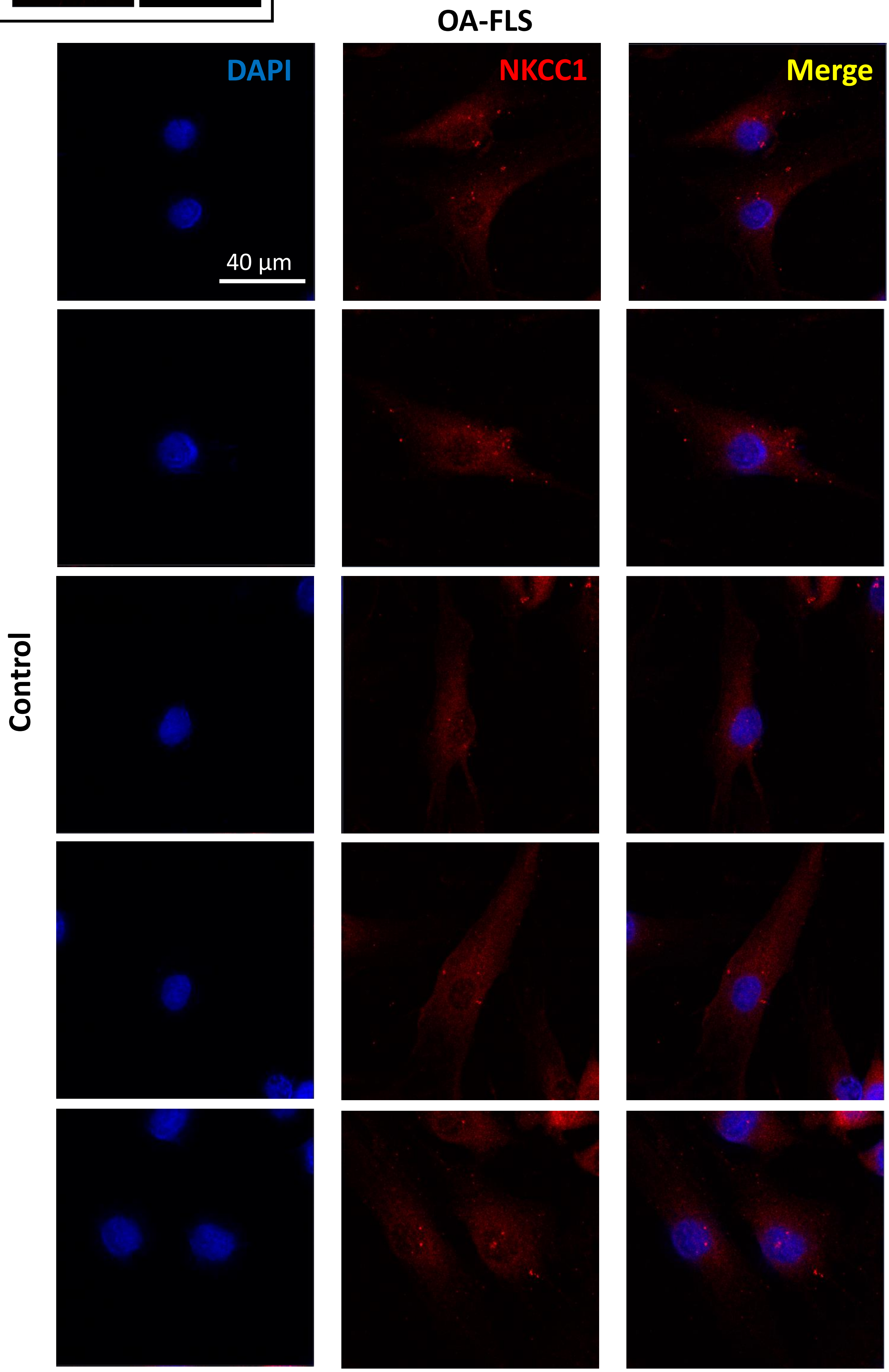

Figure 4. Ji et al.

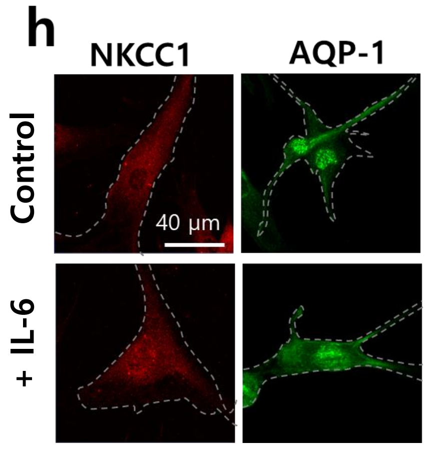

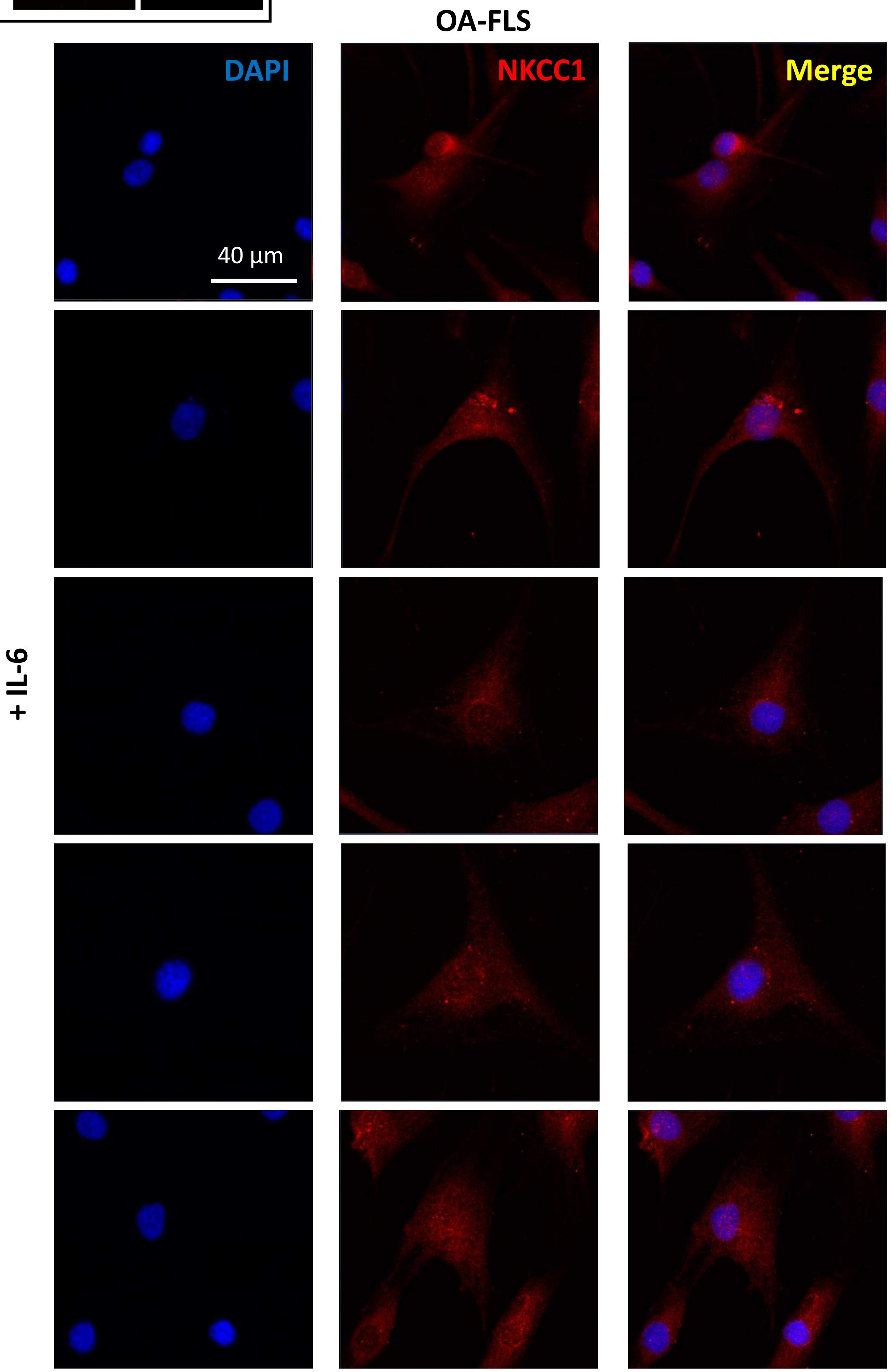

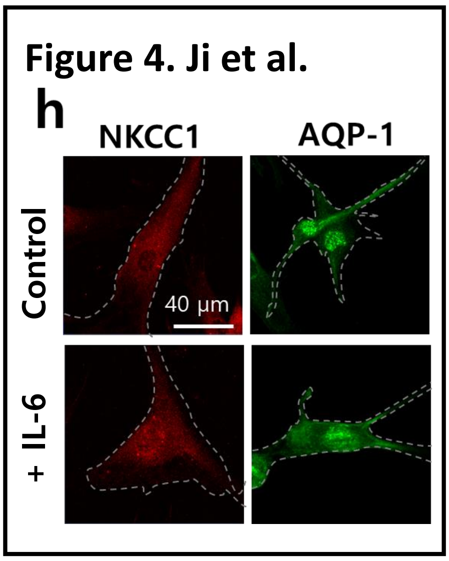

Figure 4. Ji et al.
h
NKCC1
AQP-1
Control
+ IL-6
40 μm

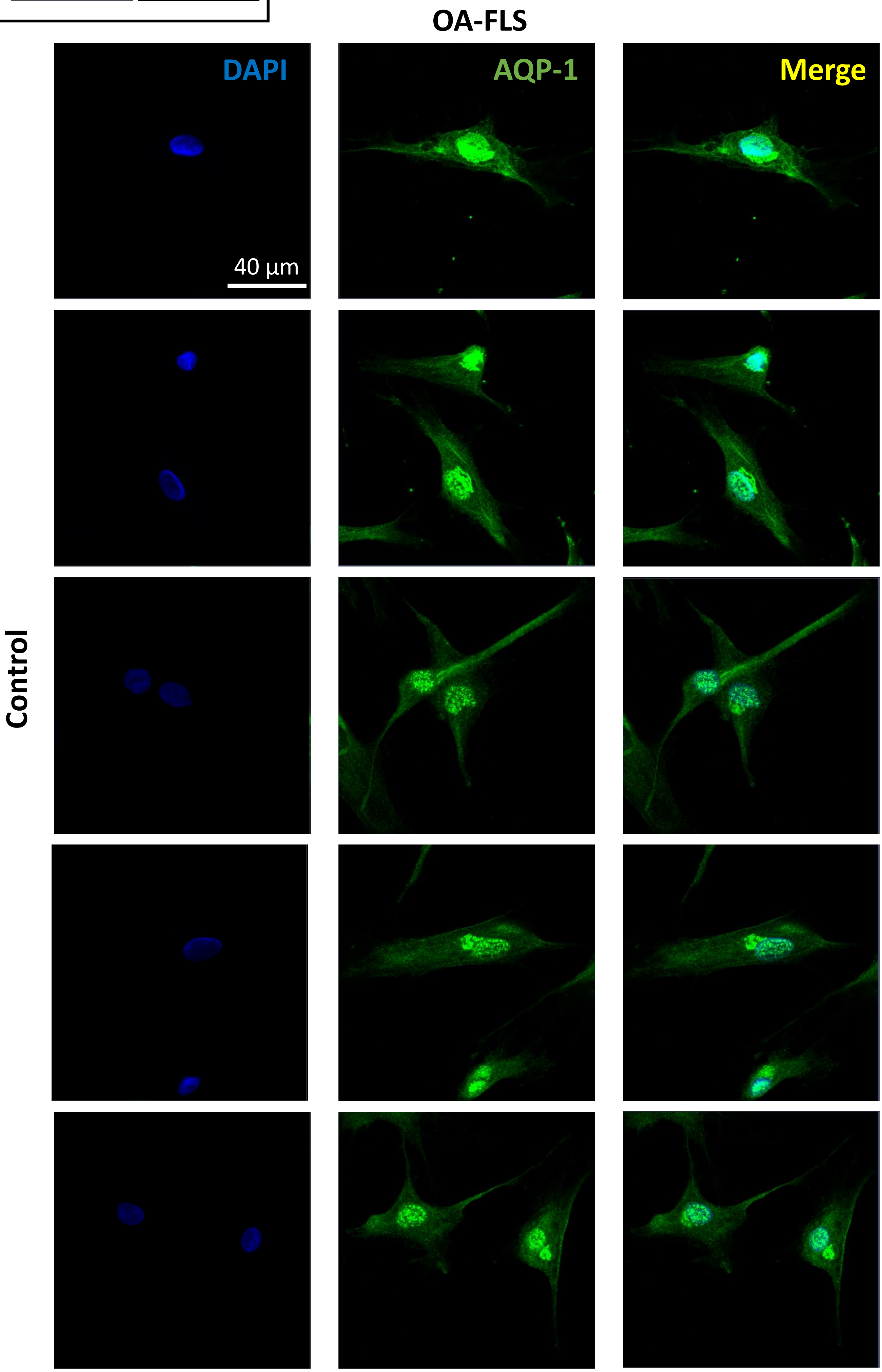

OA-FLS
DAPI
AQP-1
Merge
40 μm
Control

Figure 4. Ji et al.

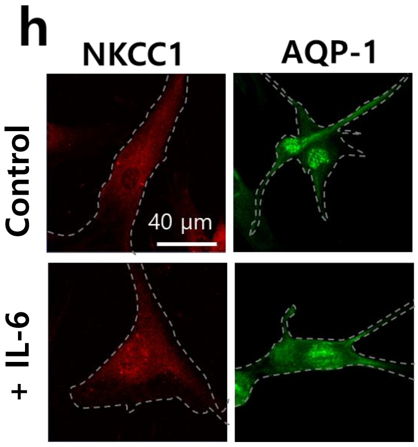

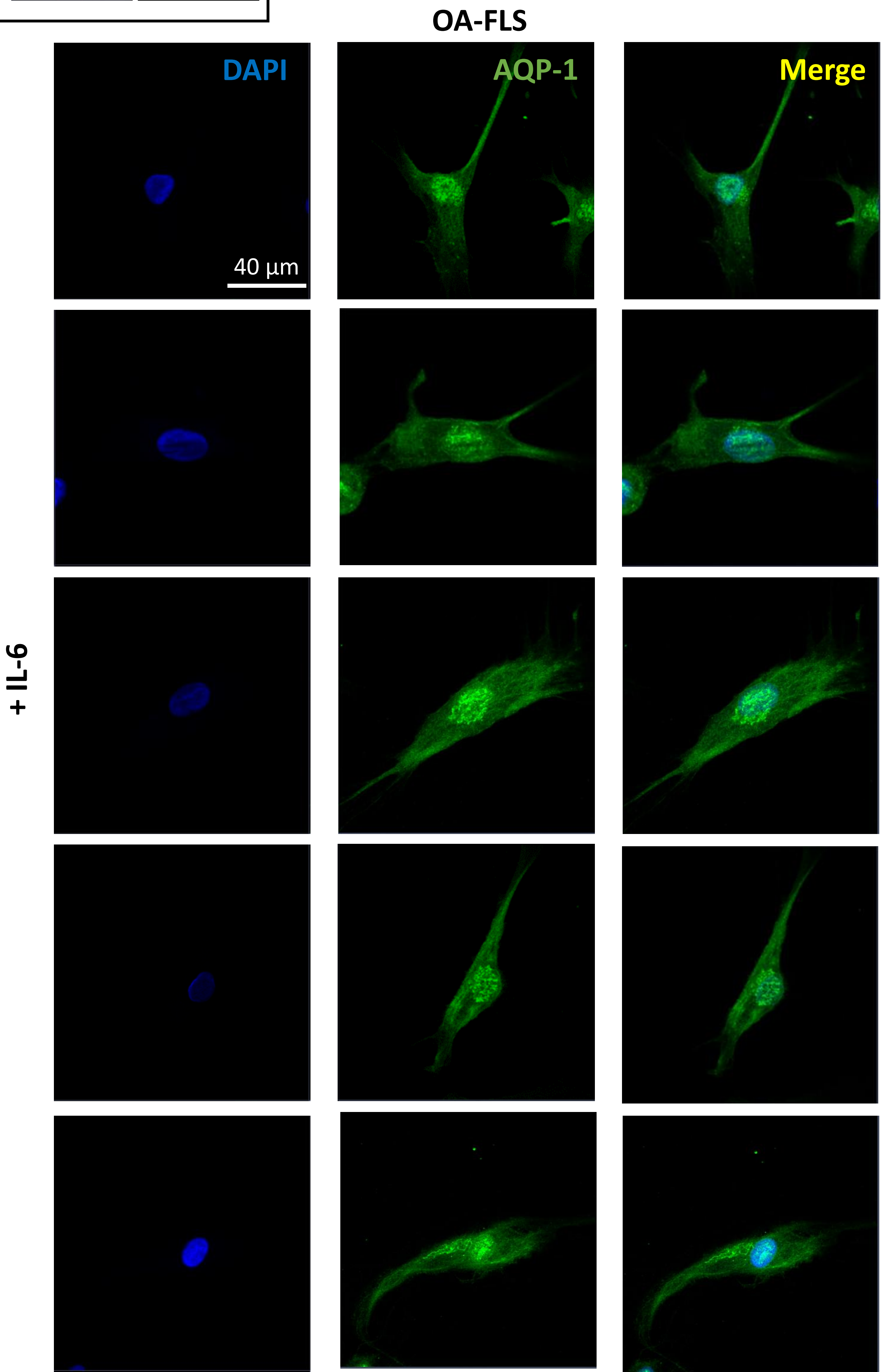

Supplement: Supplementary file 1 [file Presentation_1.pdf]
